# Exploiting natural riboswitches for aptamer engineering and validation

**DOI:** 10.1093/nar/gkac1218

**Published:** 2023-01-09

**Authors:** Michael G Mohsen, Matthew K Midy, Aparaajita Balaji, Ronald R Breaker

**Affiliations:** Department of Molecular, Cellular and Developmental Biology, Yale University, New Haven, CT 06511, USA; Howard Hughes Medical Institute, Yale University, New Haven, CT 06511, USA; Department of Molecular Biophysics and Biochemistry, Yale University, New Haven, CT 06511, USA; Department of Molecular, Cellular and Developmental Biology, Yale University, New Haven, CT 06511, USA; Department of Molecular, Cellular and Developmental Biology, Yale University, New Haven, CT 06511, USA; Howard Hughes Medical Institute, Yale University, New Haven, CT 06511, USA; Department of Molecular Biophysics and Biochemistry, Yale University, New Haven, CT 06511, USA

## Abstract

Over the past three decades, researchers have found that some engineered aptamers can be made to work well in test tubes but that these same aptamers might fail to function in cells. To help address this problem, we developed the ‘Graftamer’ approach, an experimental platform that exploits the architecture of a natural riboswitch to enhance in vitro aptamer selection and accelerate in vivo testing. Starting with combinatorial RNA pools that contain structural features of a guanine riboswitch aptamer interspersed with regions of random sequence, we performed multiplexed in vitro selection with a collection of small molecules. This effort yielded aptamers for quinine, guanine, and caffeine that appear to maintain structural features of the natural guanine riboswitch aptamer. Quinine and caffeine aptamers were each grafted onto a natural guanine riboswitch expression platform and reporter gene expression was monitored to determine that these aptamers function in cells. Additionally, we determined the secondary structure features and survival mechanism of a class of RNA sequences that evade the intended selection strategy, providing insight into improving this approach for future efforts. These results demonstrate that the Graftamer strategy described herein represents a convenient and straightforward approach to develop aptamers and validate their in vivo function.

## INTRODUCTION

Aptamers are nucleic acid structures that form binding pockets for their target ligands (1). Aptamers have diverse and increasing applications in synthetic biology (2,3), biosensing (4,5) and therapeutics (6,7). RNA aptamers are of particular interest because they can be genetically encoded and expressed in cells. Many examples of naturally occurring RNA aptamers have been discovered as components of riboswitches, which are noncoding RNA gene regulatory devices that also contain an expression platform (8,9). The expression platform manipulates downstream gene expression in response to ligand binding by the adjoining aptamer domain (10). Some riboswitches are hypothesized to be remnants of an RNA World (11,12) and are thought to have persisted in nature over billions of years (13). Thus, riboswitches provide proven architectures and mechanisms for RNAs that sense ligands and robustly regulate gene expression in complex cellular environments.

Despite the abundance and diversity of natural riboswitches, there is a pressing need for aptamers that bind additional ligands, such as drug or drug-like compounds that are both bioavailable and safe for use in humans (14,15). New aptamers for different ligands are typically developed in vitro, using techniques such as systematic evolution of ligands by exponential enrichment (SELEX) (1,16). However, the physicochemical conditions in a test tube do not match those of cells, and therefore intrinsic factors such as aptamer folding and function might differ under the two conditions (17). As a result, many aptamers developed using SELEX may not be functional in cells, even if extrinsic factors such as ligand bioavailability and biostability are adequate.

Recently, researchers have repurposed the architectures of natural riboswitch aptamers as scaffolds for the creation of aptamers for different ligands (18,19). By preserving secondary and tertiary structural elements from riboswitch aptamers, it is hoped that new aptamers derived from these preexisting scaffolds might exhibit more reliable intracellular folding (18). In one report, regions of randomized RNA nucleotides were inserted into scaffolds derived from guanine (*Bacillus subtilis xpt-pbuX*) and cyclic di-GMP-I (*Vibrio cholerae* Vc2) riboswitch aptamers (18). Using SELEX, several classes of synthetic aptamers were developed for 5-hydroxytryptophan and 3,4-dihydroxyphenylalanine (18). In another recent report, researchers utilized a scaffold based on an adenine riboswitch aptamer (*Vibrio vulnificus add*) to develop the ‘squash’ aptamer, which binds GFP-like fluorogenic dyes DFHBI-1T and DFHO (19). These examples have established that performing in vitro selection with scaffolded RNA pools can yield aptamers that retain features of the scaffold, but bind different compounds. However, the overall process would be further improved if a rapid method for verification of intracellular aptamer function were also available.

Current methods for intracellular aptamer validation, such as monitoring fluorescence intensity for fluorogenic light-up aptamers (17,19), are often specific to the aptamer of interest and not widely applicable (20). One generalizable approach entails generating fusions of an aptamer of interest and a fluorogenic aptamer, *e.g*. ‘broccoli’ (17), linked by a communication module (18,21). This process involves screening constructs with varied communication modules to identify an allosteric device in which ligand binding by the aptamer of interest gates ligand binding by the fluorogenic aptamer. Past efforts have produced in vivo sensors, which simultaneously validate both the aptamer and the allosteric function of the switching device (18,22).

**Figure 1.**
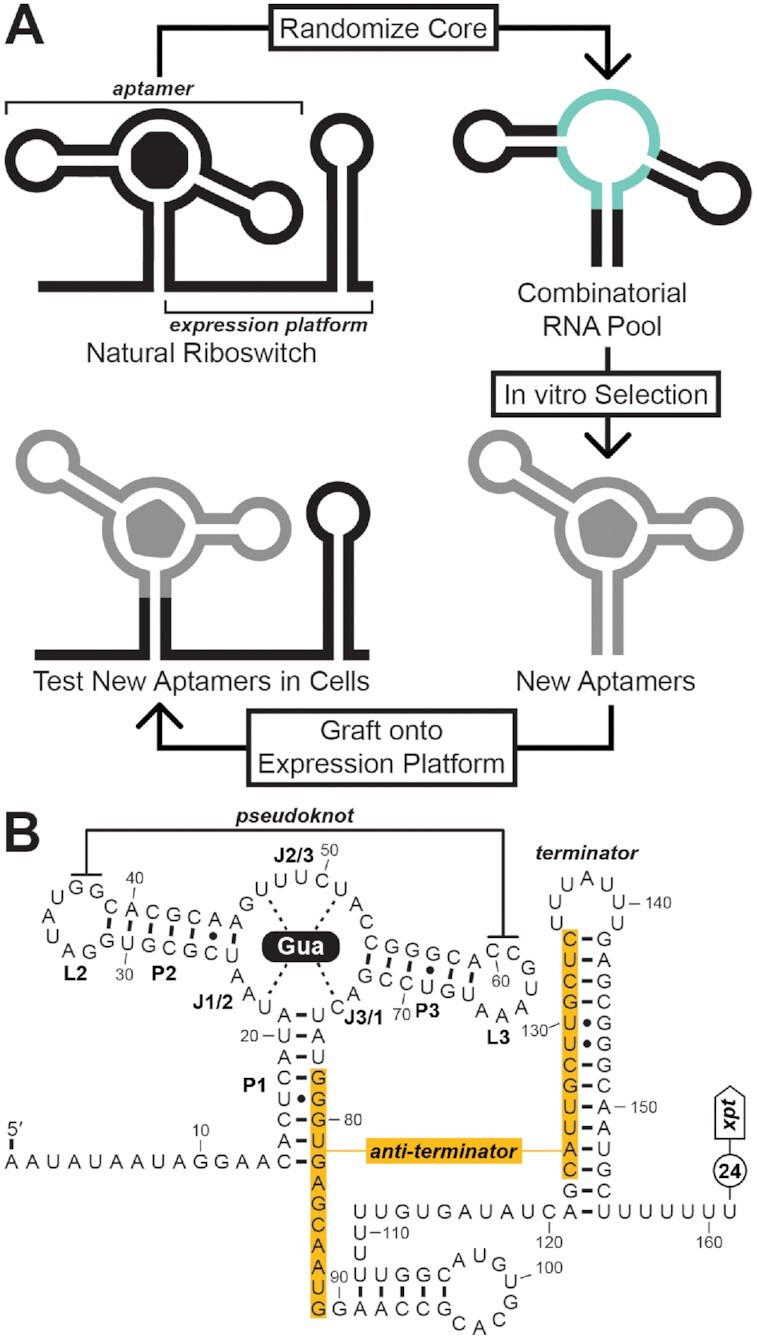
Exploiting components of a natural riboswitch to serve as a scaffold for novel aptamer development in vitro and functional validation in cells. (**A**) The Graftamer approach. The ligand-binding core of a natural riboswitch aptamer is replaced with regions of random sequence (cyan) to generate a combinatorial RNA pool. In vitro selection is performed to isolate RNA aptamers that likely retain the riboswitch aptamer scaffold but bind different compounds. These aptamers are grafted back onto the natural riboswitch expression platform and tested to validate function in cells. (**B**) The *B. subtilis xpt-pbuX* Guanine-I riboswitch is formed by a guanine-binding aptamer and an expression platform that includes an intrinsic transcription terminator stem. As an OFF switch, guanine binding by the aptamer domain presents the transcription terminator stem, which attenuates transcription of downstream genes. Nucleotides joining the three stems of the aptamer are labeled J1/2, J2/3 and J3/1 to designate the substructures they connect. When insufficient concentrations of guanine are present in the cell, an anti-terminator stem (orange) is formed, which enables expression of downstream genes.

To promote even greater confidence in the use of this process, we adapted an aptamer-expression platform fusion that is inspired by a natural system. We envisioned that newly created aptamers derived from preexisting scaffolds could be grafted back onto the expression platform of the riboswitch from which the scaffold was derived (Figure 1A, ‘Graftamer’ method). Reuse of the natural expression platform simplifies the engineering challenge and increases the probability that the resulting integrated device will regulate gene expression. Intracellular ligand binding could then be validated by installing this potential synthetic riboswitch upstream of a reporter gene and monitoring changes in gene expression within a suitable test organism. In principle, our approach could be applied to aptamers for any bioavailable compound and is widely accessible to researchers with basic molecular biology and microbiology skills.

**Figure 2.**
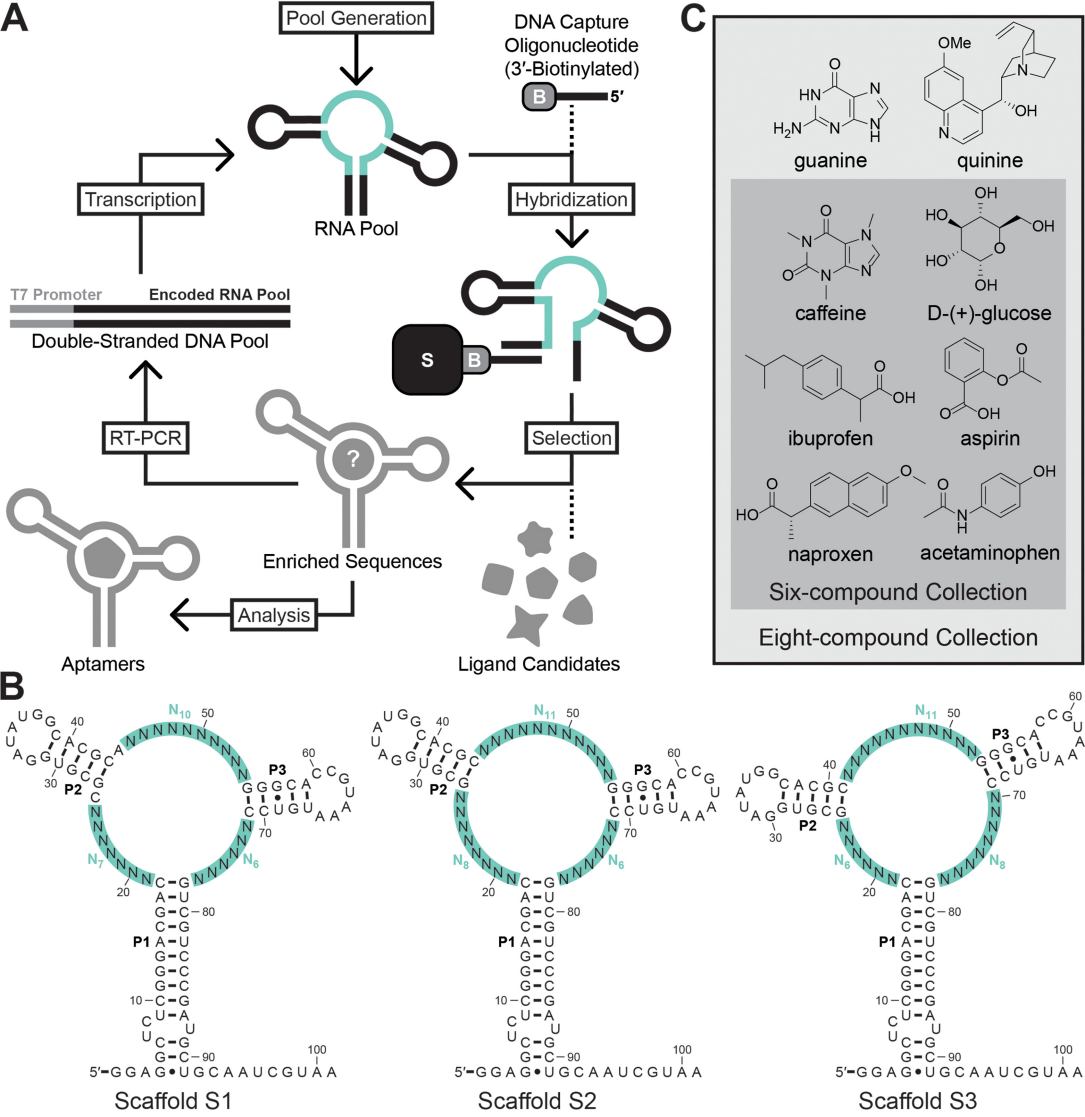
Multiplexed Capture-SELEX strategy. (**A**) The initial RNA pool is generated by in vitro transcription from oligodeoxynucleotide templates containing randomized regions. The resulting pool is annealed to a 3′-biotinylated (encircled B) DNA capture oligonucleotide and immobilized to a streptavidin-agarose (S) column. Selection is performed in multiplex with a collection of ligand candidates, wherein ligands that bind and promote the formation of P1 of the original scaffold structure are expected to be released from the column. Eluted RNAs are amplified by reverse transcription-polymerase chain reaction (RT-PCR) to yield double-stranded DNA which includes a promoter for T7 RNA polymerase. The next generation RNA pool is prepared through in vitro transcription. Iterative cycles yield highly represented candidate aptamers which are validated biochemically to determine ligand specificity. (**B**) Scaffold S1 includes structural elements (P2 and P3) from the Guanine-I riboswitch aptamer (Figure 1B). In addition to the 23 randomized nucleotides distributed among the three joining regions, the P1 stem was altered to contain a sequence that is complementary to the capture oligonucleotide and primers used for PCR amplification. In S2, C26 and A44 were also randomized. S3 is formed with some joining regions that are different in length compared to the other constructs. (**C**) The initial collection of eight ligand candidates contained various drug or drug-like compounds, plus guanine and glucose. The six-compound collection is a subset of this collection, excluding guanine and quinine.

Furthermore, we envisioned that selection could be performed in multiplex to develop aptamers for various ligand candidates simultaneously. An additional motivating factor for performing selection in multiplex is the possibility that an aptamer for a particular compound might not be present within the sequence space of a given pool (23). Considering that there are often multiple aptamer-ligand pairs that could be useful for a particular application, multiplexing provides a higher rate of success of obtaining aptamers for at least one target ligand. Multiplexing was implemented with Capture-SELEX, wherein a 3′-biotinylated DNA capture oligonucleotide is used to immobilize the RNA pool to a streptavidin-agarose column (Figure 2A) (24–27). After immobilization, a solution containing multiple ligand candidates is used to elute RNAs that bind any of these compounds.

We considered three factors when selecting a riboswitch aptamer to employ as a scaffold. First, we favored scaffolds that form tertiary contacts that could confer a three-dimensional structural framework to new aptamers that maintain this architecture. Second, we sought scaffolds that form a paired element (called a P1 stem) that encloses the entire motif and whose nucleotides also participate in expression platform folding. A P1 stem that fits this description could be exploited to compete with a capture oligonucleotide in the described selection strategy and would also provide a straightforward path to grafting new aptamers onto the original expression platform for testing in cells. Third, we considered motifs that have been naturally repurposed to bind various ligands. We hypothesized that versatile motifs which have evolved to bind diverse compounds could be further repurposed through in vitro selection to bind additional compounds with differing chemical properties. Members of the originally reported Guanine-I riboswitch class (28) employ a pseudoknot (kissing-loop structure) between loop regions L2 and L3 as well as a P1 stem that encloses the motif and, in some known examples, interplays with the expression platform (Figure 1B). This general architecture has been naturally exploited to form eight distinct riboswitch classes [Guanine-I (28), Guanine-II (29), Adenine (30), 2′-deoxyguanosine-I (2′-dG-I) (31), 2′-dG-II (32), 2′-dG-III (29), Xanthine-II (29) and 8-oxoguanine (29)] as well as several examples of engineered aptamers with three specificities [5-hydroxytryptophan (18), 3,4-dihydroxyphenylalanine (18), and GFP-like fluorogenic dyes (19)]. The established diversity of ligand binding capabilities suggests that this scaffold could be further exploited to bind additional ligands, including compounds that are both bioavailable and safe for use in humans.

For the Graftamer approach described herein, we chose to exploit components of the *B. subtilis xpt-pbuX* guanine riboswitch (Figure 1B) for creating and testing new aptamers. This riboswitch example is a highly studied and well-understood representative of the Guanine-I riboswitch class with a genetic ‘OFF’ switch function (27,32–37). Specifically, binding of guanine to the aptamer domain permits the formation of an intrinsic transcription terminator stem (38,39), thereby switching off expression of downstream genes (28).

After generating combinatorial RNA pools derived from this aptamer, we performed multiplexed in vitro selection and identified new aptamers for quinine, guanine, and caffeine that retain the main features of the natural aptamer scaffold. We then constructed quinine and caffeine riboswitches by grafting the respective aptamer onto the *B. subtilis xpt-pbuX* guanine riboswitch expression platform, in the process replacing the original guanine aptamer. DNA sequences representing these engineered riboswitches were each positioned upstream of a *lacZ* reporter gene and the resulting constructs were integrated into the genome of *B. subtilis*. Cultures containing these inserts displayed decreased reporter gene expression in the presence of the corresponding ligand, indicating that these synthetic riboswitches function as expected in cells. These results demonstrate the utility of the Graftamer approach for evolving multiple aptamers simultaneously that bind new compounds and for testing aptamer function in vivo. Moreover, this experimental pipeline is straightforward and generalizable, thereby simplifying the path to engineering and validating new aptamers and riboswitches that function in cells.

## MATERIALS AND METHODS

### Reagents

Quinine hydrochloride, guanine hydrochloride, naproxen sodium, acetaminophen, acetylsalicylic acid (aspirin) were obtained from Combi-Blocks. d-(+)-Glucose was obtained from Fisher Chemical™. Ibuprofen, caffeine, and all other chemicals were obtained from Sigma-Aldrich^®^. All chemicals were used without further purification. Taq DNA polymerase and T7 polynucleotide kinase were obtained from New England Biolabs^®^. SuperScript™ III and TURBO™ DNase I were obtained from Invitrogen™. rAPid alkaline phosphatase was obtained from Roche. RNase T1 from *Aspergillus oryzae* was obtained from Boehringer Mannheim. All commercial enzymes were used with the provided buffers and recommended conditions in accordance with the manufacturer's instructions, unless otherwise stated. T7 RNA polymerase was purified in-house. Synthetic oligodeoxynucleotides were custom ordered from either Integrated DNA Technologies (IDT) or Sigma-Aldrich^®^. Plasmids containing synthetic inserts were custom ordered from GenScript. Pierce™ Streptavidin Agarose (#20353) was obtained from Thermo Scientific™. Micro Bio-Spin™ Chromatography Columns (#7326204) were obtained from Bio-Rad. Performa DTR Gel Filtration Cartridges (#98780) were obtained from Edge Bio.

### Biological resources

*Bacillus subtilis* strain 1A1 was obtained from American Type Culture Collection (ATCC).

### In vitro selection

#### G0 pool generation

For S1-G0: First, a double-stranded DNA (dsDNA) pool template was prepared by performing overlap extension with SuperScript III reverse transcriptase (RT) in a 50 μl reaction. 120 pmol of S1-G0 Template was mixed with 180 pmol of Forward Primer in a solution containing 1 mM deoxynucleotide triphosphates (dNTPs), 10 mM dithiothreitol (DTT), and First Strand buffer (SuperScript™ III reaction buffer provided by the manufacturer). The resulting solution was heated at 90°C for 1 min to anneal the template and primer, and subsequently allowed to cool at room temperature for 3 min. 2 μl of SuperScript III was added to this solution at a final concentration of 8 units/μl. This solution was incubated at 55°C for 60 min to allow the overlap extension reaction to occur. Then, the solution was heated at 75°C for 5 min to heat-inactivate RT. Next, the DNA template was transcribed in vitro using T7 RNA polymerase. Five 100-μl transcription reactions each containing one-fifth of the volume of the overlap extension reaction (24 pmol DNA), 2 mM NTPs, transcription buffer (15 mM MgCl_2_, 2 mM spermidine, 5 mM DTT, 50 mM Tris–HCl [pH 7.5 at ∼20°C]), and T7 RNA polymerase were incubated at 37°C. After 3 h, a white precipitate (Mg_2_P_2_O_7_) was observed. The reactions were pooled and 5 μl DNase I was added. After incubating at 37°C for 10 min, the reaction was mixed with an equal volume of loading buffer (18 M urea, 20% w/v sucrose, 0.1% w/v sodium dodecyl sulfate, 0.05% w/v bromophenol blue, 0.05% xylene cyanol, 90 mM Tris, 90 mM borate, 1 mM EDTA pH 8.0). The resulting pool of RNA transcripts was purified by denaturing (8 M urea) 10% w/v polyacrylamide gel electrophoresis (PAGE) under denaturing conditions followed by precipitation in ethanol. Other G0 pools were prepared with slight modifications to this protocol.

#### Multiplexed Capture-SELEX

In Round 1, a 250 μl solution containing 120 pmol of S1-G0 RNA, 600 pmol of capture oligonucleotide, and 1x selection buffer was heated at 85°C for 1 min. The solution was allowed to cool at room temperature for ≥5 min. While the annealed RNA-capture oligonucleotide solution was cooling, a spin column was loaded with 250 μl streptavidin-agarose and the storage buffer was drained by gravity flow. To equilibrate the matrix with selection buffer, the matrix was washed six times with 250 μl selection buffer. The annealed RNA-capture oligonucleotide solution was then loaded onto the column and collected in a collection tube. The flowthrough was applied to the column 2–4 additional times, to ensure that the maximal amount of RNA was loaded onto the column. Next, the column was washed ten times with 250 μl selection buffer to remove nonspecifically bound RNA. Subsequently, 250 μl of a solution containing the eight-compound collection (100 μM each) in selection buffer was applied to the column three times. The three eluates were collected, and concentrated using a VivaSpin 10 kDa molecular weight cutoff filter. The concentrated RNA was amplified by reverse transcription-polymerase chain reaction (RT-PCR) as described below. In rounds 2–6, a 250 μl solution containing 100 pmol of the RNA pool, 500 pmol of capture oligonucleotide, and selection buffer was heated at 85–90°C for 1 min. Subsequent steps were performed without modification to the protocol described above for Round 1. In rounds 7–13, a 100-μl solution containing 40 pmol of the RNA pool, 200 pmol of capture oligonucleotide, and selection buffer and heated at 90°C for 1 min. The solution was allowed to cool at room temperature for ≥5 min. While the annealed RNA-capture oligonucleotide solution was cooling, a spin column was loaded with 100 μl streptavidin-agarose. Using an air pressure control device constructed by inserting the needle of a 1-ml syringe through a polypropylene cap that fits onto the top of the column, the storage buffer within the streptavidin-agarose matrix was drained into a collection tube. The streptavidin-agarose matrix was then washed six times with 100 μl of selection buffer. In this case, a wash was performed by adding selection buffer to the column matrix and immediately draining the solution using the air pressure control device. The annealed RNA-capture oligonucleotide solution was then loaded onto the column, and the solution was pushed through the matrix using the air pressure control device. The flowthrough was applied to the column 2–4 additional times, to ensure that the maximum amount of RNA was loaded onto the column. The column was then washed ten times with 100 μl selection buffer to remove nonspecifically bound RNA. Subsequently, 100 μl of a solution containing the eight-compound collection (100 μM each) in selection buffer was applied to the column. This solution was incubated for 2.5 min before it was drained into a collection tube using the air pressure control device. This step was repeated twice. Subsequent steps were performed without modification to the protocol described above. In rounds 14–16, the protocol was performed as described above for rounds 7–13, except that the six-compound collection was used instead of the eight-compound collection. The quinine reselection and selection with the S2 and S3 scaffolds were performed with slight modifications to this protocol (see Additional Methods section in Supplementary Data file S1).

#### Reverse transcription PCR (RT-PCR)

After performing selection, the concentrated RNA was reverse transcribed using SuperScript™ III. To the concentrated RNA solution, 1 μl of a 10 mM solution of dNTPs and 1 μl of a 2 μM Reverse Primer solution were added, along with deionized water (if necessary) up to a volume of 14 μl in a 0.6-ml tube. The solution was incubated at 65°C for 5 min, then immediately cooled on ice for at least 1 min. The tube was briefly centrifuged, then 4 μl First Strand buffer, 1 μl 0.1 M DTT, and 1 μl SuperScript™ III reverse transcriptase were added. The solution was briefly mixed by pipetting, and then incubated at 55°C for 60 min. The solution was then optionally heated at 70°C for 15 min to heat-inactivate RT (this step was not always performed, since the first step of PCR involves heating to 95°C). To perform PCR, 5 μl of this RT reaction was transferred to a 0.2-ml tube, to which was added 5 μl 10x Standard Taq Buffer, 2 μl 10 μM Forward Primer, 2 μl 10 μM Reverse Primer, 1 μl 10 mM dNTPs, 34.5 μl deionized water, and 0.5 μl Taq DNA polymerase. Between one and three 50 μl PCR reactions were set up to generate dsDNA templates for the next round of selection. The following thermal cycling program was used for PCR: initial denaturation (95°C, 2 min), denaturation (92°C, 15 s), annealing (59°C, 30 s), extension (72°C, 45 s), final extension (72°C, 2 min). The number of PCR cycles (denaturation, annealing, and extension) performed in each round of selection was determined by scout PCR or by monitoring quantitative PCR (qPCR) curves. Scout PCR was performed by removing 5-μl aliquots from a PCR reaction every 2–3 cycles. The aliquots were analyzed by agarose gel electrophoresis to determine the cycle number at which the desired product was present without overamplification. For qPCR analysis, a 2 μl from the RT reaction was mixed with 6 μl of water, 2 μl of a solution containing 4 μM each of Forward and Reverse Primers, and 10 μl of Bio-Rad iTaq Universal SYBR Green Supermix. The qPCR program used the thermal cycling parameters shown above for 25–40 cycles. The cycle number at which the resulting fluorometric curve began to plateau (after the inflection point) was noted. This number of cycles was used for PCR amplification to generate dsDNA templates for the next round of selection.

### Elution profile

Radiolabeled RNA was prepared by in vitro transcription with T7 RNA polymerase in the presence of α-^32^P-UTP. In some cases, radiolabeled RNA was prepared by performing in vitro transcription, dephosphorylating PAGE-purified RNA with alkaline phosphatase, followed by phosphorylation with T4 polynucleotide kinase in the presence of γ-^32^P-ATP. Radiolabeled RNA (∼25–50 kcpm) was mixed with unlabeled RNA (10 pmol), capture oligonucleotide (50 pmol), and selection buffer in a 100-μl solution. The solution was heated at 90°C for 1 min, after which the solution was allowed to cool at room temperature for ≥5 min. 100 μl streptavidin-agarose was added to a spin column, and the air pressure control device was used to drain the storage buffer. The column matrix was washed with selection buffer six times. The annealed RNA-capture oligonucleotide solution was then applied to the column three times, and the final flow through was collected in a collection tube (this sample is referred to as ‘Unbound RNA’). The column matrix was subsequently washed by adding selection buffer and immediately pushing the solution through. The wash step was repeated as needed until eluates reached background levels of radioactivity (typically 5–8 washes) as approximated by a Geiger counter. Each wash was collected in a collection tube (these samples are referred to as ‘Wash #N’, where N is the number of washes that have been performed). In some cases, a ‘Mock Elution’ was performed by loading 100 μl selection buffer onto the column and using the air pressure control device to incubate the matrix in selection buffer for 2.5 min before draining the solution into a collection tube. If necessary, additional washes were performed until eluates reached background levels of radioactivity. Next, a 100-μl solution containing 100 μM (unless specified otherwise) of a test compound or a collection of test compounds was loaded onto the column. This step was performed similarly to the ‘Mock Elution’ described above. After collecting the eluate, washes were performed until eluates reached background level. If desired, several tests of this nature were performed sequentially. Once all eluates were collected, 20 μl of each eluate was pipetted onto a sheet of filter paper in a grid-like pattern. The filter paper was exposed to a phosphor screen overnight. The resulting autoradiogram was produced by phosphor imaging with a Typhoon scanner. Densitometry was performed using ImageJ software.

### In-line probing

In-line probing was performed following a previously reported protocol (40). 4 μl of 5′ ^32^P-labeled RNA (∼7.5 kcpm/μl) was mixed with 1 μl of a 10× concentrated stock solution of the desired ligand. A ‘No Ligand’ sample was prepared by adding 1 μl of deionized water. For the ‘No Reaction’ sample, 4 μl of 5′ ^32^P-labeled RNA was mixed with 6 μl deionized water and 10 μl loading buffer and stored at –20°C. Additional RNA was set aside for the ‘T1’ ladder and ‘^−^OH’ ladders and stored at -20°C. The in-line probing reactions were incubated at 75°C for 1 min, then allowed to cool at room temperature for at least 5 min. 5 μl of 2× in-line probing buffer (20 mM MgCl_2_, 100 mM KCl, 50 mM Tris–HCl [pH 8.3 at ∼20°C]) was added to each sample and the reactions were incubated for approximately 48 h. On the day slated for PAGE separation of in-line samples, a T1 ladder was prepared by mixing 1 μl 5′ ^32^P-labeled RNA, 6 μl loading buffer, 1 μl reaction buffer (0.25 M sodium citrate pH 5.0), and 1 μl RNase T1. The reaction was incubated at 55°C for 15 min, after which 4 μl loading buffer and 6 μl deionized water were added to the reaction. The ^−^OH ladder was prepared by mixing 1 μl 5′ ^32^P-labeled RNA, 1 μl 0.5 M sodium bicarbonate (NaHCO_3_) pH 9.2, and 8 μl deionized water. The reaction was incubated at 90°C for 5 min, after which 10 μl loading buffer was added to the reaction. All in-line probing reactions were quenched with the addition of 10 μl loading buffer. The reactions products were separated by denaturing 10% PAGE. The gel was then dried, exposed to a phosphor screen, and imaged using a Typhoon scanner. Densitometry was performed using ImageJ or ImageQuant™ software. Representative in-line probing gels were used to estimate the reported *K*_D_ values (see Supplementary Note 1).

### Cell culture

#### Transformation of B. subtilis

A glycerol stock stored at –80°C containing *B. subtilis* 1A1 was used to inoculate 1 ml of transformation media (109 mM K_2_HPO_4_, 44 mM KH_2_PO_4_, 3.4 mM trisodium citrate, 760 μM MgSO_4_, 14 mM NaSO_4_, 50 μM FeCl_3_, 2 μM MnSO_4_, 0.4% w/v glucose, 0.2% w/v glutamate, 50 μg/ml tryptophan). The inoculated media was incubated overnight at 37°C with agitation at 220 rpm. The next day, 1 μg of plasmid was added to the culture, which was incubated at 37°C with agitation at 220 rpm for 40 min. Next, 1 ml of lysogeny broth (LB) and 1 μl 0.1 mg/ml chloramphenicol was added and the culture was incubated at 37°C with agitation at 220 rpm for 45 min. The culture was transferred to a 1.6-ml tube and pelleted by centrifugation. Most of the supernatant was aspirated, leaving only 100–200 μl. The pellet was resuspended in the remaining supernatant, and the entire mixture was plated on an LB agar plate supplemented with 5 μg/ml chloramphenicol and incubated at 37°C overnight. The next day, a transformant colony was picked with an inoculating loop and patched on a new LB agar plate supplemented with 100 μg/ml spectinomycin. The same inoculating loop was used to re-streak the transformant on a new LB agar plate supplemented with 5 μg/ml chloramphenicol. 4–8 transformants were screened in this way. Valid clones grew in the presence of chloramphenicol, but not in the presence of spectinomycin.

#### Monitoring lacZ expression in B. subtilis

A glycerol stock stored at –80°C containing *B. subtilis* with the desired genomic integration was streaked onto an LB plate supplemented with 5 μg/ml chloramphenicol and incubated overnight at 37°C. The next day, a single colony was inoculated in modified glucose minimal media (MGMM—1× Spizizen salts, 0.5% glucose, 0.5 mM CaCl_2_, 2.5 mM MgCl_2_, 5 μM MnCl_2_, 50 μg/ml tryptophan, 50 μM FeSO_4_) and incubated overnight at 37°C with agitation at 220 rpm. The following day, the overnight culture was diluted 1:10 into fresh MGMM supplemented with the desired concentration of quinine, 100 μg/ml X-Gal and 5 μg/ml chloramphenicol. To prepare concentrated stock solutions of quinine, quinine HCl was dissolved in DMSO. Thus, samples without quinine were mock treated with DMSO. Cultures were monitored visually until differential intensity in blue color was observed. For *B. subtilis* cultures carrying the Tonic construct with various concentrations of quinine (Figure 5B, left), the cultures were incubated at room temperature without agitation for 6 days. For comparison of Tonic, Tonic-M1, and Tonic-M2 constructs, the cultures were incubated at 37°C with agitation at 220 rpm for 48 h. Culture tubes were photographed using the Camera app on a Google Pixel 6 smartphone.

#### Miller assays

A glycerol stock stored at –80°C containing *B. subtilis* with the desired genomic integration was streaked onto an LB plate supplemented with 5 μg/ml chloramphenicol and incubated overnight at 37°C. The next day, a single colony was inoculated in MGMM and incubated overnight at 37°C with agitation at 220 rpm. The following day, overnight cultures were diluted 1:10 into 1 ml fresh MGMM supplemented with the desired concentration of 10 μl 100 mM caffeine or 10 μl H_2_O (mock treatment). Three technical replicates were prepared for each experimental condition. The cultures were incubated at 37°C with agitation at 220 rpm for 5 h. 100 μl aliquots were pipetted into a 96-well plate and OD_600_ was measured. 100 μl uninoculated media was used as a blank. 50 μl of permeabilization buffer (100 mM Tris [pH 7.8 at ∼20°C], 32 mM Na_2_HPO_4_, 8 mM DTT, 8 mM cyclohexanediaminetetraacetic acid, 4% Triton X-100) supplemented with 0.75 mg/ml lysozyme was added to each well. After waiting 15 min, 50 μl of 4 mg/ml *ortho*-nitrophenyl-β-galactoside (ONPG) was added to each well. A plate reader was used to measure OD_420_ at 1-min intervals over a 2 h period while incubating at 28°C. The blank OD_420_ reading (from uninoculated media) was subtracted from the OD_420_ reading of each sample at every timepoint. Specific β-galactosidase activity was calculated by determining the slope (OD_420_/min) of the linear portion of the OD_420_ versus time curve for each sample and dividing this value by the corresponding OD_600_ reading. Statistical analysis was performed with a t-test (two-tailed distribution, two sample equal variance).

### Next generation sequencing of selection pools

50 ng of dsDNA for a given pool was submitted to the Yale Center for Genomic Analysis. Illumina NovaSeq was used to perform next-generation sequencing (ca. 40 million reads). Paired-end reads were sequenced with a read length of 150 base pairs.

### Bioinformatics

Python scripts from Supplementary Data File S2 were used to analyze sequencing data. The python script *toTally* was executed to merge paired-end reads and tally the merged reads. *toTally* uses bbmerge (41) (part of the bbmap suite of tools) to merge the paired-end reads. The output of *toTally* is a tab-separated values (.tsv) file that lists all unique reads in ranked order with the following information: RNA sequence, number of reads, percent abundance, and rank within the pool. This tallied output file was analyzed to identify highly represented sequences within the pool. The *selfishCluster* script was used to identify sequence clusters that are considered to be part of the same class. This script uses a similar algorithm to that of the Perl-based FASTAptamer (42) bioinformatic toolkit, although ours was independently derived and is written in Python. *selfishCluster* accepts user-queried rank(s) within the pool and parses through the tallied output file to identify the top 1000 sequences that belong to this class. For example, if ranks 1 and 2 are queried, *selfishCluster.py* will first parse through the tallied output file and compare each sequence to the reference sequence by pairwise alignment. If a sequence is within 90% similarity of each other (excluding user-provided constant regions), then this sequence will be added to the cluster. This will occur repeatedly until 1000 sequences are found, or until the script has reached the end of the file. Then, the script will perform the same analysis starting with the rank 2 sequence. If any queried sequences belong to the same class, the script will skip the analysis of the latter sequences and state that this sequence has already been placed into another cluster. *selfishCluster* outputs the results of this analysis to the standard output, including the number of sequences belonging to each cluster as well as the percent abundance within the pool that is represented by each analyzed cluster. Additionally, *selfishCluster* outputs a separate fasta (.fa) file containing all sequences that belong to each cluster that was queried. These fasta files were analyzed by CMfinder (43) to generate Stockholm (.sto) files containing putative structural motifs information. In some cases, the consensus secondary structure motifs outputted by CMfinder was adjusted manually to conform to a structural model supported by in-line probing analysis. R2R (44) was then used to first generate a weighted consensus and then to draw the associated consensus secondary structure models for each class.

## RESULTS AND DISCUSSION

### Multiplexed Capture-SELEX and analysis of highly enriched sequences

We designed scaffold S1 by randomizing 23 nucleotides within the *B. subtilis xpt-pbuX* Guanine-I riboswitch aptamer and adapting the P1 stem to be compatible with our Capture-SELEX design (Figure 2B). In accordance with a previous design (18), these 23 nucleotides comprised all positions in the joining regions, including those known to directly interact with guanine based on crystallographic data (33). P1 was also altered from the natural sequence to enable hybridization to a DNA capture oligonucleotide that has previously been used successfully for Capture-SELEX (26). The starting pool of RNA (generation 0, or G0) based on scaffold S1 could include 7 × 10^13^ distinct sequences. 120 pmol of this ‘S1-G0’ pool was used in the initial selection, which is sufficient to encompass all possible sequences.

We prepared a compound collection that included several over-the-counter drugs, glucose, and guanine (Figure 2C). Guanine was included as a control ligand candidate that should yield aptamers because the pool is expected to include sequences that match the original natural aptamer consensus model. Selection was performed by heating and cooling the RNA pool in the presence of a 5-fold molar excess of the DNA capture oligonucleotide, loading the annealed DNA-RNA molecules onto a streptavidin-agarose column, stringently washing to remove nonspecifically bound RNA, and eluting with a solution containing the eight-compound collection (100 μM each). RNAs that eluted from the column were amplified by reverse transcription and polymerase chain reaction (RT-PCR), followed by in vitro transcription with T7 RNA polymerase.

**Figure 3.**
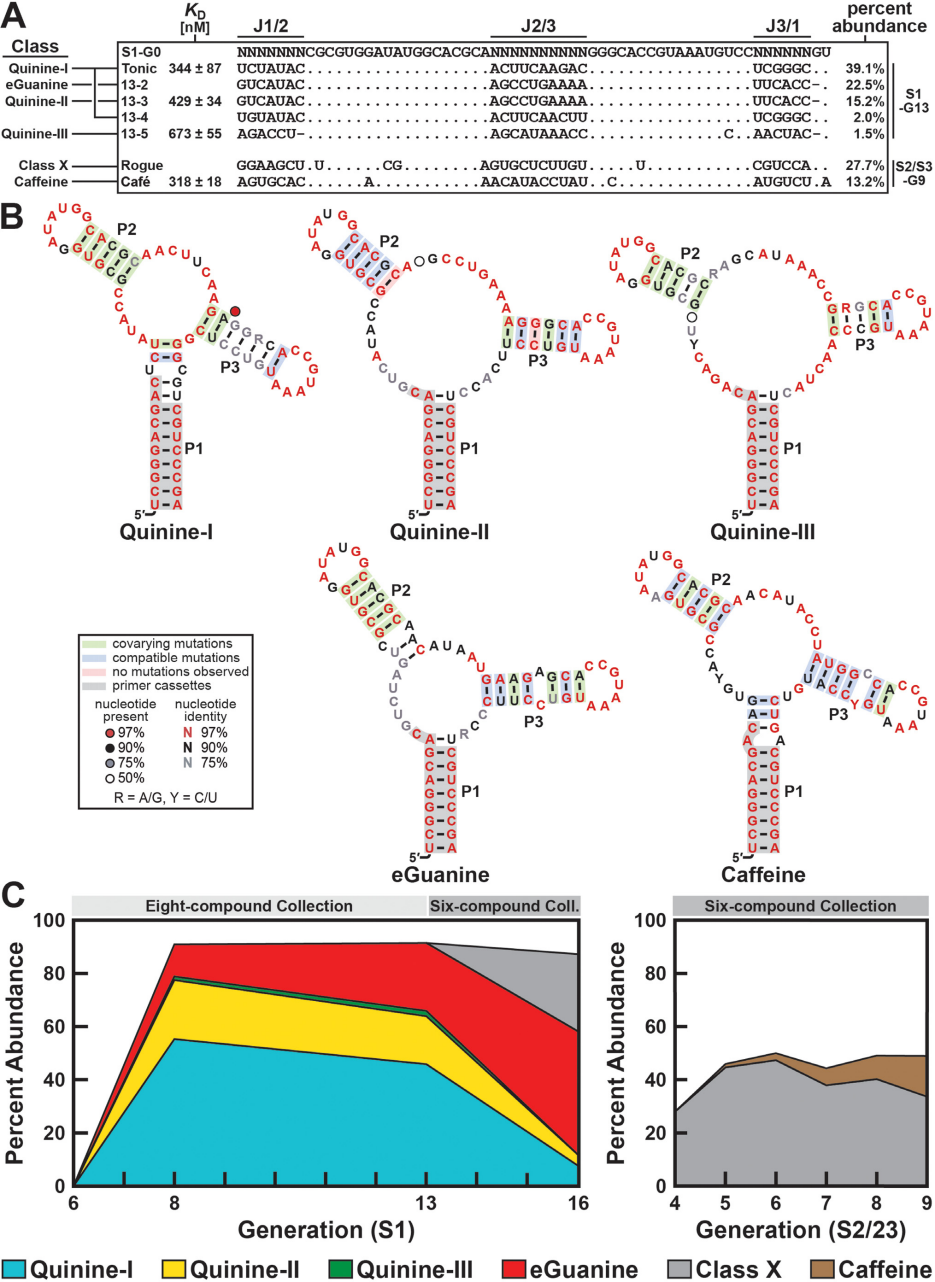
Computational analysis of sequenced RNA pools reveals highly enriched sequences and distinct aptamer classes. (**A**) Sequence alignment (excluding primer-binding regions) and percent abundance of highly represented sequences as identified by computational analysis of the indicated sequenced pools. *K*_D_ values are estimated by in-line probing analysis (*n* = 1) using multiple reactions sampling a range of ligand concentrations. Standard error values are derived from goodness of fit to a sigmoidal curve. (**B**) Consensus sequence and secondary structure models of experimentally validated aptamer classes. Models for the Quinine-I through -III classes were generated from analysis of sequencing data obtained from the G5 quinine reselection pool. Models for the eGuanine and Caffeine classes were generated using data from the S1-G16 and S2/S3-G9 pools, respectively. Green shading represents instances of covariation. (**C**) Tracking sequence classes over generations of in vitro selection. The eight-compound collection (Figure 2C) was used in selection rounds 1–13 with the S1 scaffold. The six-compound collection (Figure 2C) was used in selection rounds 14–16 with the S1 scaffold and rounds 1–9 with the S2 and S3 scaffolds. Sequenced pools are indicated by the corresponding generation numbers labeled on the x-axis.

After 13 rounds of selection, an elution profile (see Methods section) revealed that the resulting S1-G13 pool yields a strong elution signal only with quinine and guanine (Supplementary Figure S1A). This pool was subjected to next-generation sequencing (NGS, ca. 40 million reads) and analyzed computationally (see Methods section for details). We identified the five most highly represented sequences, 13-1 through -5 (Figure 3A), for further analysis. Ligand selectivity was established for each of the five RNAs by performing an elution profile with solutions containing quinine, guanine, and then the other six compounds combined. Each of these sequences selectively eluted with either quinine (13-1, -3, -4 and -5) or guanine (13-2) (Supplementary Figure S1B). The best-performing quinine aptamer (13-1) was named ‘Tonic’ (Figure 3A).

### Biochemical and bioinformatic analysis of quinine and guanine aptamers

The result of each elution profile experiment to determine ligand selectivity was interpreted qualitatively. We then further validated ligand binding affinity using in-line probing assays, which exploit the natural instability of RNA to monitor RNA folding changes mediated by ligand binding (40,45). Representative in-line probing data with the Tonic aptamer reveals modulation in the intensity of 5′ ^32^P-labeled RNA cleavage products only when incubated with quinine (Supplementary Figure S2). Titration with quinine revealed that modulation was dose-dependent (Supplementary Figure S2C), and a *K*_D_ of 344 ± 87 nM was estimated for this interaction by quantifying and plotting the band intensities from three modulating sites (Supplementary Figure S2D).

The 13-4 sequence differs from that of Tonic at only four positions, and both RNAs elute with quinine. These sequences were considered members of a single aptamer class named Quinine-I. In contrast, quinine-responsive sequences 13-3 and 13-5 each carry many more sequence differences compared to the Quinine-I class and compared to each other. Therefore, we considered these to be representatives of distinct aptamer classes Quinine-II and Quinine-III, respectively. 13-2 is considered a representative of the ‘eGuanine’ (engineered Guanine) class. In-line probing analysis (Supplementary Figures S3–S6) was carried out for each of these aptamers to estimate the *K*_D_ values summarized in Figure 3A.

Because of the original pool design and the inclusion of guanine in the compound collection used for selection, the emergence of RNAs with sequences that conform to the natural guanine aptamer consensus was possible. Although a guanine-responsive RNA class is represented among the most abundant RNAs from the selected pool, its core sequence (Figure 3B) does not conform to the consensus for natural guanine riboswitch aptamers (9,29). Upon analysis of the sequenced pools, we do identify RNA representatives that carry the consensus for the natural guanine aptamer, but these sequences are exceedingly rare. Furthermore, we cannot rule out the possibility that they are derived from molecular contamination rather than from the original selection pool.

There are several possible reasons why representatives of the natural aptamer class were not more abundant in the selected population. For example, the natural guanine aptamer is formed using ten highly conserved nucleotides in the regions joining its three stems, whereas the guanine aptamer identified in the present study appears to have no more than four. Thus, alternative aptamer structures that are simpler and more frequently represented in the original RNA pool might be more likely to dominate the final selected RNA population. In contrast, rarely represented aptamer classes might be lost early in the selection due to simple stochastic events that prevented their release and amplification in early selection cycles. Furthermore, selected RNAs must bind a target ligand and alter their structures in a manner that releases the RNA from the capture oligonucleotide. It is likely that some aptamer classes release from the column more effectively than others when binding ligand, meaning that some reasonable aptamer classes might be lost due to their failure of ligand binding to trigger release.

Based on sequence analyses and in-line probing assay results, each of these aptamers appears to retain the key structural features of the original guanine riboswitch aptamer scaffold, including the pseudoknot between loops L2 and L3 (Supplementary Figures S2–S6). Reduced spontaneous cleavage is observed at putative base-paired regions of L2 and L3, as expected if these regions are prevented from forming an in-line geometry (45). An exception is the internucleotide linkage following the U nucleotide in L2 that resides immediately upstream of the two G nucleotides involved in the kissing loop. This same in-line probing product band pattern is also observed with the *B. subtilis xpt-pbuX* guanine riboswitch aptamer (28), and occurs because this U nucleotide is locked in a partial in-line conformation (33). These observations provide evidence that the new aptamers also exploit this pseudoknot architecture.

To produce an artificial phylogeny for each of the three classes of quinine aptamers, we performed a reselection using only quinine instead of the collection of ligand candidates. Representative examples for Quinine-I, Quinine-II, and Quinine-III (Tonic, 13-3 and 13-5, respectively) were chosen for this reselection. The sequence of each of these aptamers was mutagenized at 6% degeneracy, excluding the primer-binding regions. The resulting three mutagenized DNA templates were mixed in an equimolar ratio. Primer extension and in vitro transcription were subsequently performed to generate a G0 reselection pool. Five rounds of selection were performed with 1 μM quinine and the resulting G5 pool was sequenced. The sequencing data was analyzed computationally (see Methods section for details) to identify the top 1000 members of class Quinine-I, the top 140 members of class Quinine-II (only 140 unique sequences met this criteria), and the top 1000 members of class Quinine-III. The Quinine-I class contained both Tonic and 13-4, as expected. For each of these classes, CMfinder (43) was used to generate consensus sequence and secondary structure models, which were then drawn with R2R (44) (Figure 3B). Notably, the structure predicted by consensus models is consistent with in-line probing data.

After having identified three quinine aptamer classes and one guanine aptamer class, we remained interested in pursuing aptamers for other compounds from our initial collection. We anticipated that by excluding quinine and guanine from the compound collection and performing additional rounds of selection starting from the S1-G13 pool, aptamers for other compounds would quickly come to dominate the selected population. After performing three additional rounds of selection with the six-compound collection (Figure 2C) and analyzing the sequencing data of the resulting S1-G16 pool, we found that quinine aptamers indeed had rapidly dropped in abundance as expected (Figure 3C). However, two surprising results were observed. First, a previously unidentified sequence had reached 22.6% of the population. This sequence failed to display specific elution with any of the six ligand candidates from the collection (Supplementary Figure S7). We suspected that this sequence employed a ligand-independent survival mechanism (*vide infra*: Identification and Characterization of a Class of Selfish Sequences). Because this sequence appeared to evade our intended selection strategy, we named this sequence ‘Rogue’ (Figure 3A). Second, guanine aptamer 13-2 had become the prevailing sequence despite guanine being removed from the collection (Figure 3C). We suspect that 13-2 may employ a mixed survival strategy that includes the (intended) guanine-dependent release from the capture oligonucleotide as well as an (unintended) ligand-independent mechanism.

### A caffeine aptamer is uncovered by its enrichment signature

To outmaneuver selfish sequences, we initiated a new line of selection in which we added more stringent washing steps. We also took this opportunity to adjust the design of scaffold S1. We recognized that the aptamers examined from the S1-G13 pool all contain a C–A mismatch in P2 as dictated by the original design of the S1 pool (C26, A44). We hypothesized that this forced mismatch might pose undesired limitations on the length of P2 and consequently the structural diversity of the pool. To address this, we designed S2, wherein these nucleotides were randomized to provide the possibility for base pairing (Figure 2B). We also designed an additional scaffold (S3) in which the first (N_8_) and third (N_6_) random regions were swapped to further increase structural diversity (Figure 2B). These two scaffolds were combined in a 1:1 ratio to prepare a new S2/S3-G0 pool. Nine rounds of selection were performed with the six-compound collection (Figure 2C). Analysis of sequencing data from the S2/S3-G9 pool revealed that the selfish sequence Rogue was the most highly represented sequence in this pool, comprising 28.2% of the population (Figure 3A). It is statistically improbable that this sequence has an independent origin in this selection, and so we reasoned that its presence was most likely due to contamination from the previous selection (see Supplementary Note 2).

Despite the prevalence of a selfish sequence in the S2/S3 selection line, we reasoned that a structure-switching aptamer that elutes in the presence of its target ligand should eventually be able to outcompete the selfish, slow-leaking strategy (*vide infra*) employed by Rogue. To find these sequences, we analyzed sequencing data from the G4–G9 pools. We found that after just four generations, Rogue comprised 24.5% of the population, providing additional support for our contamination hypothesis (Figure 3C). However, analysis also revealed a strong enrichment profile for a candidate sequence that comprised 13.5% of the pool after nine generations (Figure 3C, right panel). An elution profile revealed that this sequence exhibited specificity for caffeine over the other five compounds tested (Supplementary Figure S1B) and was further validated by in-line probing to be a caffeine aptamer (Supplementary Figure S8). This sequence, which we named ‘Café’ (Figure 3A), is the most represented sequence of the Caffeine-I class of caffeine aptamers (Figure 3B). The consensus model for Caffeine-I was generated by analyzing sequencing data from the S2/S3-G9 pool.

Interestingly, we were unable to identify any highly represented sequences that originated from scaffold S3 (Figure 2B). Perhaps rare aptamers exist from this population, but their performance characteristics were insufficient to promote robust increases in abundance compared to the selfish RNA contaminant and the S2-derived representatives. We also considered the possibility that caffeine aptamer Café may have derived from S1 because it contains the C-A mismatch that is a design characteristic of scaffold S1. Computational analysis revealed that Café was the 923rd ranked sequence in the S1-G16 pool, comprising 0.002% of the pool. Although it is impossible to discern definitively, this suggests that Café may have originated from the S1 selection as well.

### Testing aptamer function in cells

The aptamers that arose from our selection experiments have P1 stems that differ from that of the parent guanine riboswitch aptamer. To create engineered riboswitches that closely conform to the guanine riboswitch representative that inspired the design of the original selection constructs, we replaced P1 of the Tonic aptamer with the P1 of the *B. subtilis xput-pbuX* guanine riboswitch aptamer. Only the two base pairs nearest to the three-way junction were maintained to avoid ligand binding disruption in the event that these positions are important for ligand recognition. In-line probing with this RNA revealed that the Tonic aptamer retains quinine binding function and successfully differentiates quinine from guanine when grafted onto the guanine riboswitch context (Supplementary Figure S9). Grafting the aptameric core sequences of 13-3 and Café onto the guanine riboswitch context also altered the specificity from guanine to quinine and caffeine, respectively (Supplementary Figures S10 and S11).

**Figure 4.**
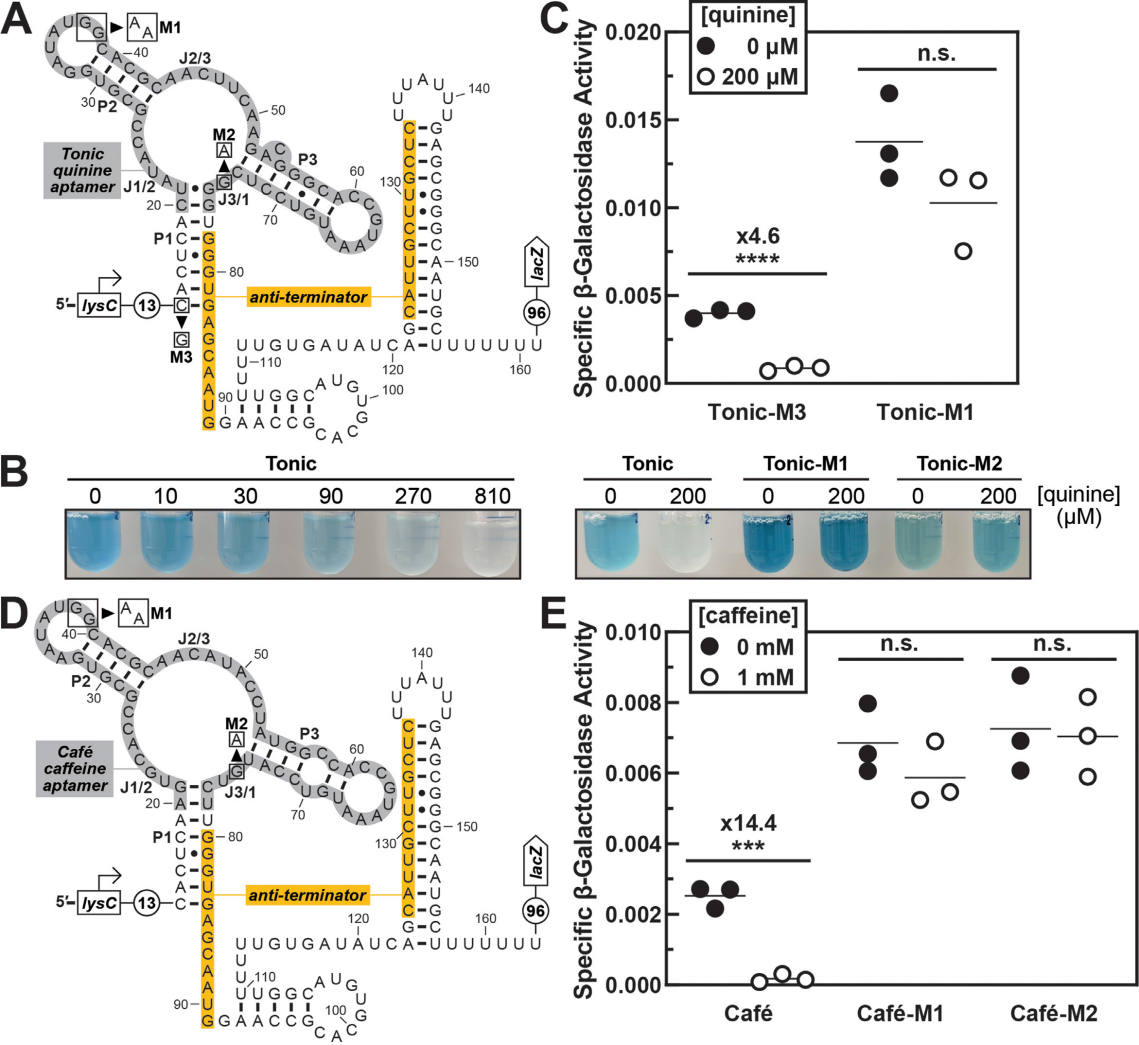
In vivo function of engineered riboswitches reveals that Tonic and Café aptamers function in cells. (**A**) A synthetic quinine riboswitch constructed by grafting the quinine aptamer Tonic onto the expression platform of the natural *B. subtilis xpt-pbuX* Guanine-I riboswitch. Constructs Tonic-M1 and -M2 contain nucleotide changes that disrupt ligand binding. Construct Tonic-M3 contains a nucleotide change which shortens the length of the P1 stem. (**B**) Images of riboswitch-reporter fusion assays conducted with *B. subtilis* cells carrying the indicated constructs. β-galactosidase reporter activity was assessed by evaluating the intensity of blue color. (**C**) Results of the Miller assay performed with *B*. subtilis cells carrying the indicated constructs, cultured in media supplemented with the indicated concentrations of quinine. Each point represents data from one technical replicate, and the thin line indicates the mean. ****P < 0.0001. (**D**) A synthetic caffeine riboswitch constructed by grafting the caffeine aptamer Café onto the expression platform of the natural *B. subtilis xpt-pbuX* Guanine-I riboswitch. Constructs Café-M1 and -M2 contain nucleotide changes that disrupt ligand binding. (**E**) Results of a Miller assay performed with *B*. subtilis cells carrying the indicated constructs, cultured in media supplemented with the indicated concentrations of caffeine. Each point represents data from one technical replicate, and the thin line indicates the mean.***P < 0.001.

Next, we tested the in vivo function of the quinine aptamer Tonic in bacterial cells. Tonic was grafted onto the expression platform of the *B. subtilis xpt-pbuX* guanine riboswitch, replacing the original guanine aptamer and producing a synthetic ‘quinine riboswitch’ (Figure 4A). This quinine riboswitch was positioned downstream of a constitutive promoter (*B. subtilis lysC*) and upstream of the open reading frame for a *lacZ* reporter gene (Figure 4A) within a pDG1661 vector. This construct was integrated into *B. subtilis* strain 1A1 genome at the *amyE* locus. *B. subtilis* cultures containing this insert were grown at various concentrations of quinine (0–810 μM), revealing a progressive decrease in the intensity of blue color with higher concentrations of ligand (Figure 4B). Quinine-binding by the Tonic aptamer domain is expected to permit the formation of the terminator stem, which attenuates transcription and reduces expression of *lacZ*. The results of the reporter assays support the conclusion that quinine aptamer Tonic retains quinine-binding function in *B. subtilis* cells and that the engineered riboswitch functions as a genetic OFF switch.

*B. subtilis* cells are known to grow without inhibition on agar plates containing up to 1.5% w/v (2 mM) quinine sulfate (46). However, the possibility remained that the observed reporter gene assay results are triggered by some other unexpected effects of quinine on cells. To confirm that quinine-dependent reduction in gene expression is due to aptamer-mediated regulation of transcription termination, we designed two mutant riboswitch constructs that exhibit reduced affinity for quinine. Variant Tonic-M1 contains a GG to AA mutation that disrupts the formation of a putative pseudoknot (Figure 4A, construct M1). Variant Tonic-M2 (Figure 4A, construct M2) carries a G to A mutation at nucleotide 74, which forms J3/1. This G74A mutation was prepared because this position was identified as highly conserved upon analysis of the sequencing data (Figure 3B). These constructs were evaluated by in-line probing, and both exhibited reduced quinine binding in vitro (Supplementary Figure S9). This result was recapitulated in vivo, as it was observed that *B. subtilis* cultures containing these mutant constructs retain high reporter gene expression when quinine concentrations are high (Figure 4B). Thus, the visible decrease in blue intensity associated with increasing quinine concentration is likely due to quinine binding by the aptamer domain and subsequent transcription terminator formation by the expression platform of the synthetic riboswitch.

Notably, *B. subtilis* cultures containing the Tonic-M1 variant construct exhibit increased intensity of blue color compared to the other constructs examined. It has been shown that the pseudoknot between L2 and L3 is important for preorganizing the aptamer domain to form a ligand binding pocket as the riboswitch is transcribed (35). Our results with the Tonic-M1 construct indicate that pseudoknot formation might be important for our quinine riboswitch as well. Disrupting pseudoknot formation results in a strongly disabled riboswitch, perhaps because the aptamer is unable to pre-organize to form a ligand binding pocket. Failure to form the pseudoknot might favor formation of the anti-terminator stem and allow a greater number of full-length transcripts to accumulate. Pseudoknot-disruptive variants like Tonic-M1 may thus represent a general strategy for producing control constructs for any new aptamers generated from the guanine riboswitch platform. This further simplifies the route to testing new aptamers in vivo. Control constructs are readily available to verify that any changes in reporter gene expression are indeed caused by the riboswitch, and not unexpected effects from the ligand.

Perhaps surprisingly, it took 2–6 days for *B. subtilis* cultures containing the Tonic and Tonic-M2 constructs to develop the blue color pictured in Figure 4B (see Methods section for details), indicating low activity for these constructs even in the ON state. This phenomenon is corroborated by in-line probing data (Supplementary Figure S2 and S9), which suggest that Tonic is well-folded in the absence of quinine and that modulation in the presence of the quinine is relatively modest. As a result, while the quinine riboswitch does alter gene expression in response to quinine, time is needed to observe the effects. Nonetheless, we attempted to quantitate reporter activity of *B. subtilis* cells carrying these constructs with a Miller assay, but we were unable to detect any signal over background for the conditions tested (Supplementary Figure S12). To increase the ON-state activity of the Tonic construct, we generated variant Tonic-M3 (Figure 4A), which contains a C14G mutation that effectively shortens the length of P1. We hypothesized that this mutation would facilitate the formation of the anti-terminator stem during transcription, thus increasing reporter activity in the ON state. We conducted a Miller assay with *B. subtilis* cells carrying the Tonic-M3 construct and we observed that ON-state activity was increased to a detectable range, as expected (Figure 4C). We also observed a 4.6-fold decrease in gene expression with Miller assay when *B. subtilis* cells containing this construct were cultured in the presence of 200 μM quinine (Figure 4C), providing further evidence that the quinine aptamer Tonic functions in cells.

We also produced a caffeine riboswitch by grafting the caffeine aptamer Café onto the guanine riboswitch expression platform (Figure 4D), as with the quinine riboswitch described above. *B. subtilis* cells containing this insert were cultured in the presence or absence of 1 mM caffeine. After 5 h, specific β-galactosidase activity was measured by Miller assay. Cultures grown in media supplemented with 1 mM caffeine exhibit a 14.4-fold decrease in specific β-galactosidase activity under the conditions tested (Figure 4E). This substantial decrease in reporter gene expression is consistent with our expectations that this synthetic caffeine riboswitch construct should function as a genetic OFF switch. We then tested variants Café-M1 and Café-M2, which contain mutations that disrupt pseudoknot formation or a mutation to a highly conserved nucleotide identified by bioinformatics, respectively. Cells containing these variant switches no longer respond to 1 mM caffeine (Figure 4E), providing further evidence that the result is due to the expected caffeine riboswitch function. Similarly, these results reveal that the Café caffeine aptamer is functional inside a living cell.

### Identification and characterization of a class of selfish sequences

**Figure 5.**
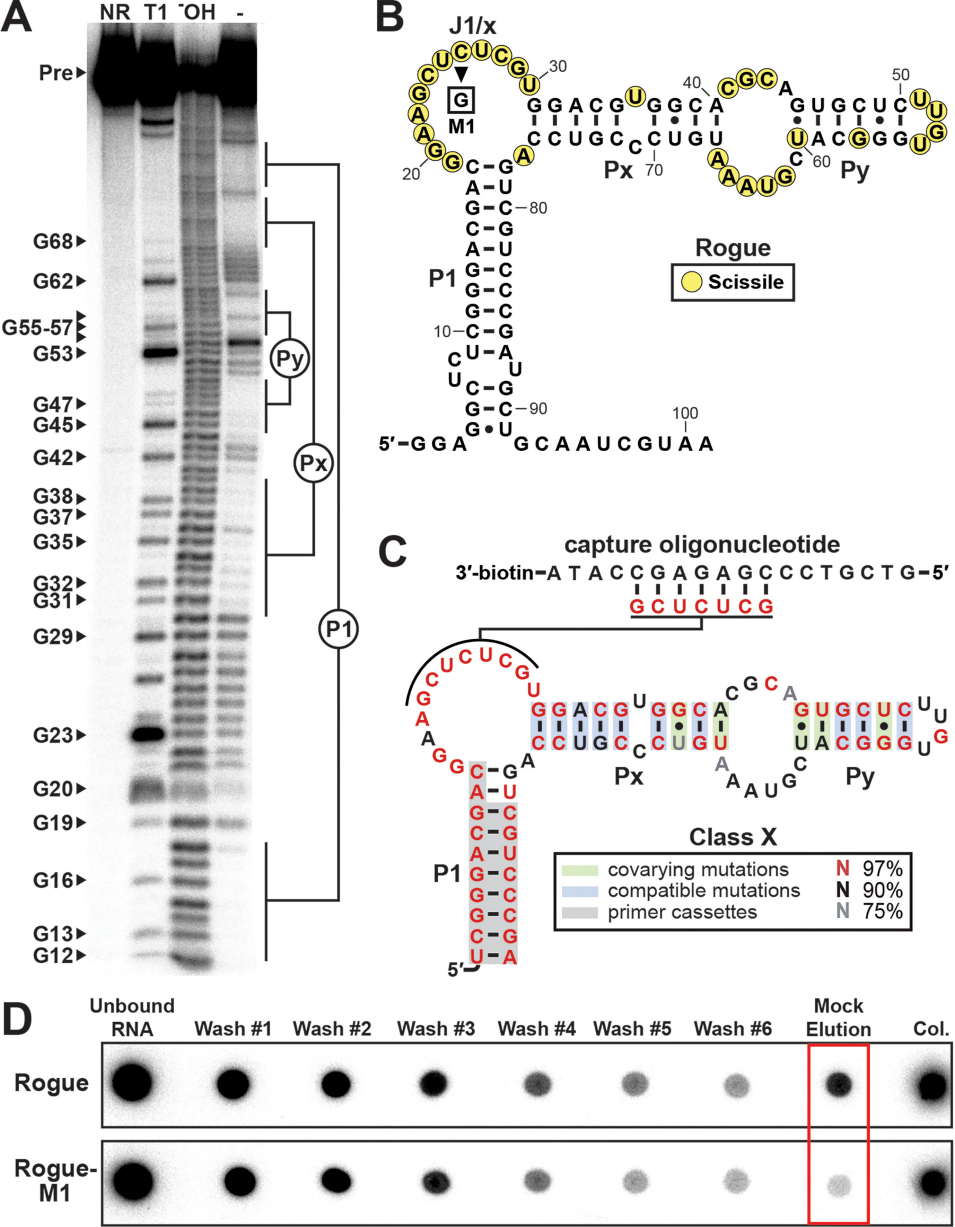
Characterization of Class X, a highly enriched class of RNA sequences with an unintended survival strategy. (**A**) In-line probing analysis of 5′ ^32^P-labeled Rogue reveals an alternative structure that does not adopt the main features of the guanine riboswitch aptamer scaffold. (**B**) Secondary structure model of Rogue as determined by in-line probing analysis. Rogue-M1 contains a C26G mutation relative to the original Rogue sequence. (**C**) Consensus sequence and secondary structure model of Class X. Green shading indicates instances of covariation. A highly conserved (>97%) stretch of seven contiguous nucleotides in the J1/x region is complementary to the capture oligonucleotide. (**D**) Comparative elution profile analysis reveals a decreased amount of eluted RNA after a 2.5-min incubation (mock elution) for the Rogue-M1 variant (red box).

The selfish sequence Rogue does not undergo structural modulation with any of the compounds as determined by in-line probing assays (Supplementary Figure S13). Moreover, the pattern of spontaneous cleavage products observed from in-line probing reactions is not consistent with the desired guanine riboswitch aptamer scaffold (Figure 5A and B). We identified several other highly represented sequences in the S1-G16 pool that appear to be from the same class as Rogue, and we generated a consensus model for this class using CMfinder and R2R (Figure 5C). Because members of this class do not elute with any of the compounds, this class was named Class X. We noted that Rogue contains seven contiguous nucleotides that are complementary to a region of the capture oligonucleotide. These seven nucleotides are colored red in our consensus model for Class X (Figure 5C), indicating that they are conserved in > 97% of examples. In-line probing analysis of Rogue (Supplementary Figure S13) and bioinformatic analysis of Class X (Figure 5C) reveal that these seven nucleotides occur in a region that is single-stranded. We hypothesized that sequences belonging to this class might alternatively hybridize to the capture oligonucleotide through these seven nucleotides in addition to the intended capture oligonucleotide binding site. If true, this base-pairing could be transient and allow RNAs to slowly leak off the column during selection. This slow but continuous leaking could serve as a mechanism by which representatives of this class populated the collection of selected RNAs and thus propagated to the next round of selection. We tested this hypothesis by introducing a C26G mutation in Rogue (Figure 5B, construct Rogue-M1), producing a mismatch between the center of this region and the capture oligonucleotide. As expected, non-specific elution was reduced with this Rogue-M1 mutant relative to the original Rogue (Figure 5D), providing additional evidence for this survival mechanism. We also observed a slight decrease in the amount of RNA remaining in the column matrix at the end of the elution profile, which is expected if one of the binding modes were abolished.

## CONCLUDING REMARKS

Capture-SELEX has several advantages over ‘conventional SELEX’ that make it an attractive strategy for in vitro selection of small molecule binding aptamers (47). In conventional SELEX, the ligand candidate is linked to a solid support matrix and the desired RNA molecules (or other nucleic acids) are separated based on their ability to bind the ligand candidate (1,47). This strategy restricts the scope of ligand candidates to those containing a subset of functional groups that can be covalently modified with a linker to a solid support. Ligand candidates used in Capture-SELEX are not constrained by this limitation. Ligand candidates are also free in solution, negating any steric effects from a solid support and increasing potential binding modes for the RNA-ligand complex. Although these advantages are also true of homogeneous selection methods such as allosteric selection (23,48), Capture-SELEX can be technically less demanding and thus perhaps more scalable.

However, recent reports have highlighted the issue of nonspecific background elution observed in Capture-SELEX (49–51). We have elucidated the molecular mechanism of a class of selfish sequences denoted Class X, which likely survive by hybridizing to the capture oligonucleotide in an unintended, transient mode and slowly leaking off the column. We anticipate that this survival mechanism is likely to arise in any Capture-SELEX experiment. We have shown that it is still possible to identify true aptamers despite the presence of highly enriched selfish molecules. However, prevalent selfish molecules may require additional rounds of selection to permit the enrichment of aptamers, adding potentially unnecessary excess labor.

To avoid this burden, we propose the following strategy for future Capture-SELEX experiments. After an RNA pool has become sufficiently enriched by in vitro selection (typically after 8–12 generations) and highly represented sequences are appropriately analyzed, a new RNA pool should be designed to initiate any additional lines of selection. New primers should be designed that do not amplify members of the previous selection experiments, and the primer-binding sites in the new pool design should be modified accordingly. With this precaution, selfish molecules from a previous selection will be less likely to contaminate a new selection line.

Capture-SELEX has been described as a selection strategy for structure-switching aptamers (24). Typically, RNA or DNA is bound to a capture oligonucleotide through hybridization of 12–15 contiguous base pairs. In principle, RNA or DNA must undergo a structural change upon binding a ligand to release from the capture oligonucleotide. We were skeptical that the free energy of binding a small molecule would be sufficient to denature 12–15 contiguous base pairs. Recently, Heemstra and coworkers tested a kanamycin A aptamer that had been previously developed using Capture-SELEX and did not observe a structure-switching event in their assay (51,52). This finding agrees with our opinion that Capture-SELEX may not select for structure-switching aptamers as initially proposed. We speculate that sequences that are fully hybridized to the capture oligonucleotide may not be able to elute at all. Instead, sequences that ‘learn’ to bind transiently to the capture oligonucleotide with fewer base pairs can survive to the next round of selection, either by quickly releasing in the presence of a target ligand (aptamers) or slowly releasing over time (selfish molecules).

In summary, we have exploited the architecture of a natural guanine riboswitch to engineer new aptamers and validate their function in cells. Using a multiplexed Capture-SELEX scheme, we developed new aptamers for quinine, caffeine, and guanine that contain the guanine riboswitch aptamer scaffold. We also uncovered a selfish class of RNA molecules that evaded the intended selection strategy with a slow-leaking mechanism. We then constructed synthetic riboswitches that independently respond to quinine or caffeine by grafting each of the corresponding aptamers onto the expression platform of a guanine riboswitch. These synthetic riboswitches exhibit decreased reporter gene expression in response to their respective ligands in *B. subtilis* cultures, thus demonstrating that these aptamers function in cells. The described Graftamer approach is comprehensive and straightforward, permitting the isolation of novel ligand-binding RNAs that can be readily validated in cells.

In this study, we have demonstrated how the architecture of a natural guanine riboswitch can be exploited to create synthetic aptamers and riboswitches. Results described herein and elsewhere (18,19,29–32) indicate that the natural guanine aptamer employs an exceptionally malleable scaffold. However, it is possible that no solution may exist within this structural space for some target ligands. Analogous experiments could be applied using the structural scaffolds of other natural riboswitches to expand this strategy beyond the guanine aptamer and its associated expression platform. This adaptation could broaden the diversity of aptamers recovered and enable a wider range of gene expression changes when engineered aptamers are integrated with expression platforms. In this manner, synthetic riboswitches constructed from these aptamers could be directly employed as designer gene regulatory devices, with diverse applications in synthetic biology and biosensing. With modifications that would allow these constructs to interface with eukaryotic cellular machinery—instead of controlling a terminator stem, the aptamer could regulate the activity of a self-cleaving ribozyme (53–55) or a splice site (56,57)—these devices could also be developed for applications in human gene therapy.

## DATA AVAILABILITY

Code used for analysis of sequencing data is contained in Supplementary File S2 (.zip).

## Supplementary Material

gkac1218_Supplemental_Files

## References

[1] Ellington A.D. , Szostak J.W. In vitro selection of RNA molecules that bind specific ligands. Nature. 1990; 346:818–822. 1697402 10.1038/346818a0

[2] Khalil A.S. , Collins J.J. Synthetic biology: applications come of age. Nat. Rev. Genet. 2010; 11:367–379. 20395970 10.1038/nrg2775 PMC2896386

[3] Wrist A. , Sun W., Summers R.M. The theophylline aptamer: 25 years as an important tool in cellular engineering research. ACS Synth. Biol. 2020; 9:682–697. 32142605 10.1021/acssynbio.9b00475

[4] Song S. , Wang L., Li J., Fan C., Zhao J. Aptamer-based biosensors. TrAC - Trends Anal. Chem. 2008; 27:108–117.

[5] McConnell E.M. , Nguyen J., Li Y. Aptamer-Based biosensors for environmental monitoring. Front. Chem. 2020; 8:434. 32548090 10.3389/fchem.2020.00434 PMC7272472

[6] Lee J.F. , Stovall G.M., Ellington A.D. Aptamer therapeutics advance. Curr. Opin. Chem. Biol. 2006; 10:282–289. 16621675 10.1016/j.cbpa.2006.03.015

[7] Keefe A.D. , Pai S., Ellington A. Aptamers as therapeutics. Nat. Rev. Drug Discov. 2010; 9:537–550. 20592747 10.1038/nrd3141 PMC7097324

[8] Nahvi A. , Sudarsan N., Ebert M.S., Zou X., Brown K.L., Breaker R.R. Genetic control by a metabolite binding mRNA. Chem. Biol. 2002; 9:1043–1049. 12323379 10.1016/s1074-5521(02)00224-7

[9] Mccown P.J. , Corbino K.A., Stav S., Sherlock M.E., Breaker R.R. Riboswitch diversity and distribution. RNA. 2017; 23:995–1011. 28396576 10.1261/rna.061234.117 PMC5473149

[10] Mandal M. , Breaker R.R. Gene regulation by riboswitches. Nat. Rev. Mol. Cell Biol. 2004; 5:451–463. 15173824 10.1038/nrm1403

[11] Benner S.A. , Ellington A.D., Tauer A. Modern metabolism as a palimpsest of the RNA world. Proc. Natl. Acad. Sci. U.S.A. 1989; 86:7054–7058. 2476811 10.1073/pnas.86.18.7054 PMC297992

[12] Chen X. , Li N., Ellington A.D. Ribozyme catalysis of metabolism in the RNA world. Chem. Biodivers. 2007; 4:633–655. 17443876 10.1002/cbdv.200790055

[13] Breaker R.R. Riboswitches and the RNA World. Cold Spring Harb. Perspect. Biol. 2012; 4:a003566. 21106649 10.1101/cshperspect.a003566 PMC3281570

[14] Mou H. , Zhong G., Gardner M.R., Wang H., Wang Y.W., Cheng D., Farzan M. Conditional regulation of gene expression by Ligand-Induced occlusion of a MicroRNA target sequence. Mol. Ther. 2018; 26:1277–1286. 29567311 10.1016/j.ymthe.2018.02.021 PMC5993935

[15] Spöring M. , Finke M., Hartig J.S. Aptamers in RNA-based switches of gene expression. Curr. Opin. Biotechnol. 2020; 63:34–40. 31811992 10.1016/j.copbio.2019.11.008

[16] Tuerk C. , Gold L. Systematic evolution of ligands by exponential enrichment: RNA ligands to bacteriophage T4 DNA polymerase. Science. 1990; 249:505–510. 2200121 10.1126/science.2200121

[17] Filonov G.S. , Moon J.D., Svensen N., Jaffrey S.R. Broccoli: rapid selection of an RNA mimic of green fluorescent protein by fluorescence-based selection and directed evolution. J. Am. Chem. Soc. 2014; 136:16299–16308. 25337688 10.1021/ja508478x PMC4244833

[18] Porter E.B. , Polaski J.T., Morck M.M., Batey R.T. Recurrent RNA motifs as scaffolds for genetically encodable small-molecule biosensors. Nat. Chem. Biol. 2017; 13:295–301. 28092358 10.1038/nchembio.2278 PMC5310984

[19] Dey S.K. , Filonov G.S., Olarerin-George A.O., Jackson B.T., Finley L.W.S., Jaffrey S.R. Repurposing an adenine riboswitch into a fluorogenic imaging and sensing tag. Nat. Chem. Biol. 2022; 18:180–190. 34937909 10.1038/s41589-021-00925-0 PMC8967656

[20] Espah Borujeni A. , Mishler D.M., Wang J., Huso W., Salis H.M. Automated physics-based design of synthetic riboswitches from diverse RNA aptamers. Nucleic Acids Res. 2016; 44:1–13. 26621913 10.1093/nar/gkv1289 PMC4705656

[21] Soukup G.A. , Breaker R.R. Nucleic acid molecular switches. Trends Biotechnol. 1999; 17:469–476. 10557159 10.1016/s0167-7799(99)01383-9

[22] Paige J.S. , Nguyen-Duc T., Song W., Jaffrey S.R. Fluorescence imaging of cellular metabolites with RNA. Science. 2012; 335:1194. 22403384 10.1126/science.1218298 PMC3303607

[23] Koizumi M. , Soukup G.A., Kerr J.N.Q., Breaker R.R. Allosteric selection of ribozymes that respond to the second messengers cGMP and cAMP. Nat. Struct. Biol. 1999; 6:1062–1071. 10542100 10.1038/14947

[24] Nutiu R. , Li Y. In vitro selection of structure-switching signaling aptamers. Angew. Chem. Int. Ed. 2005; 44:1061–1065. 10.1002/anie.200461848 15643624

[25] Lauridsen L.H. , Doessing H.B., Long K.S., Nielsen A.T. A Capture-SELEX strategy for multiplexed selection of RNA aptamers against small molecules. Methods Mol. Biol. 2018; 1671:291–306. 29170966 10.1007/978-1-4939-7295-1_18

[26] Yang K.A. , Pei R., Stojanovic M.N. In vitro selection and amplification protocols for isolation of aptameric sensors for small molecules. Methods. 2016; 106:58–65. 27155227 10.1016/j.ymeth.2016.04.032 PMC4981533

[27] Boussebayle A. , Groher F., Suess B. RNA-based Capture-SELEX for the selection of small molecule-binding aptamers. Methods. 2019; 161:10–15. 30953759 10.1016/j.ymeth.2019.04.004

[28] Mandal M. , Boese B., Barrick J.E., Winkler W.C., Breaker R.R. Riboswitches control fundamental biochemical pathways in *Bacillus subtilis* and other bacteria. Cell. 2003; 113:577–586. 12787499 10.1016/s0092-8674(03)00391-x

[29] Dhakal S.H. , Panchapakesan S.S.S., Slattery P., Roth A., Breaker R.R. Variants of the guanine riboswitch class exhibit altered ligand specificities for xanthine, guanine, or 2′-deoxyguanosine. Proc. Natl. Acad. Sci. U.S.A. 2022; 119:e2120246119. 35622895 10.1073/pnas.2120246119 PMC9295807

[30] Mandal M. , Breaker R.R. Adenine riboswitches and gene activation by disruption of a transcription terminator. Nat. Struct. Mol. Biol. 2004; 11:29–35. 14718920 10.1038/nsmb710

[31] Kim J.N. , Roth A., Breaker R.R. Guanine riboswitch variants from Mesoplasma florum selectively recognize 2′-deoxyguanosine. Proc. Natl. Acad. Sci. U.S.A. 2007; 104:16092–16097. 17911257 10.1073/pnas.0705884104 PMC1999398

[32] Weinberg Z. , Nelson J.W., Lönse C.E., Sherlock M.E., Breaker R.R. Bioinformatic analysis of riboswitch structures uncovers variant classes with altered ligand specificity. Proc. Natl. Acad. Sci. U.S.A. 2017; 114:E2077–E2085. 28265071 10.1073/pnas.1619581114 PMC5358364

[33] Batey R.T. , Gilbert S.D., Montange R.K. Structure of a natural guanine-responsive riboswitch complexed with the metabolite hypoxanthine. Nature. 2004; 432:411–415. 15549109 10.1038/nature03037

[34] Serganov A. , Yuan Y.R., Pikovskaya O., Polonskaia A., Malinina L., Phan A.T., Hobartner C., Micura R., Breaker R.R., Patel D.J. Structural basis for discriminative regulation of gene expression by adenine- and guanine-sensing mRNAs. Chem. Biol. 2004; 11:1729–1741. 15610857 10.1016/j.chembiol.2004.11.018 PMC4692365

[35] Gilbert S.D. , Stoddard C.D., Wise S.J., Batey R.T. Thermodynamic and kinetic characterization of Ligand binding to the Purine Riboswitch aptamer domain. J. Mol. Biol. 2006; 359:754–768. 16650860 10.1016/j.jmb.2006.04.003

[36] Kim J.N. , Breaker R.R. Purine sensing by riboswitches. Biol. Cell. 2008; 100:1–11. 18072940 10.1042/BC20070088

[37] Gilbert S.D. , Love C.E., Edwards A.L., Batey R.T. Mutational analysis of the purine riboswitch aptamer domain. Biochemistry. 2007; 46:13297–13309. 17960911 10.1021/bi700410g PMC2556308

[38] Wilson K.S. , Von Hippel P.H Transcription termination at intrinsic terminators: the role of the RNA hairpin. Proc. Natl. Acad. Sci. U.S.A. 1995; 92:8793–8797. 7568019 10.1073/pnas.92.19.8793 PMC41053

[39] Yarnell W.S. , Roberts J.W. Mechanism of intrinsic transcription termination and antitermination. Science. 1999; 284:611–615. 10213678 10.1126/science.284.5414.611

[40] Regulski E.E. , Breaker R.R. In-line probing analysis of riboswitches. Methods Mol. Biol. 2008; 419:53–67. 18369975 10.1007/978-1-59745-033-1_4

[41] Bushnell B. , Rood J., Singer E. BBMerge – Accurate paired shotgun read merging via overlap. PLoS One. 2017; 12:e0185056. 29073143 10.1371/journal.pone.0185056 PMC5657622

[42] Alam K.K. , Chang J.L., Burke D.H. FASTAptamer: a bioinformatic toolkit for high-throughput sequence analysis of combinatorial selections. Mol. Ther. - Nucleic Acids. 2015; 4:e230. 25734917 10.1038/mtna.2015.4 PMC4354339

[43] Yao Z. , Weinberg Z., Ruzzo W.L. CMfinder - a covariance model based RNA motif finding algorithm. Bioinformatics. 2006; 22:445–452. 16357030 10.1093/bioinformatics/btk008

[44] Weinberg Z. , Breaker R.R. R2R - software to speed the depiction of aesthetic consensus RNA secondary structures. BMC Bioinformatics. 2011; 12:3. 21205310 10.1186/1471-2105-12-3 PMC3023696

[45] Soukup G.A. , Breaker R.R. Relationship between internucleotide linkage geometry and the stability of RNA. RNA. 1999; 5:1308–1325. 10573122 10.1017/s1355838299990891 PMC1369853

[46] Antika L.D. , Triana D., Ernawati T. Antimicrobial activity of quinine derivatives against human pathogenic bacteria. IOP Conf. Ser. Earth Environ. Sci. 2020; 462:012006.

[47] Ruscito A. , DeRosa M.C. Small-molecule binding aptamers: selection strategies, characterization, and applications. Front. Chem. 2016; 4:14. 27242994 10.3389/fchem.2016.00014 PMC4861895

[48] Roth A. , Breaker R.R. Selection in vitro of allosteric ribozymes. Methods Mol. Biol. 2004; 252:145–164. 15017047 10.1385/1-59259-746-7:145

[49] Lyu C. , Khan I.M., Wang Z. Capture-SELEX for aptamer selection: a short review. Talanta. 2021; 229:122274. 33838776 10.1016/j.talanta.2021.122274

[50] Feagin T.A. , Maganzini N., Soh H.T. Strategies for creating structure-Switching aptamers. ACS Sensors. 2018; 3:1611–1615. 30156834 10.1021/acssensors.8b00516

[51] Sanford A.A. , Rangel A.E., Feagin T.A., Lowery R.G., Argueta-Gonzalez H.S., Heemstra J.M. RE-SELEX: restriction enzyme-based evolution of structure-switching aptamer biosensors. Chem. Sci. 2021; 12:11692–11702. 34659704 10.1039/d1sc02715h PMC8442683

[52] Stoltenburg R. , Nikolaus N., Strehlitz B. Capture-SELEX: selection of DNA aptamers for aminoglycoside antibiotics. J. Anal. Methods Chem. 2012; 2012:415697. 23326761 10.1155/2012/415697 PMC3544269

[53] Tang J. , Breaker R.R. Rational design of allosteric ribozymes. Chem. Biol. 1997; 4:453–459. 9224568 10.1016/s1074-5521(97)90197-6

[54] Felletti M. , Stifel J., Wurmthaler L.A., Geiger S., Hartig J.S. Twister ribozymes as highly versatile expression platforms for artificial riboswitches. Nat. Commun. 2016; 7:2–9. 10.1038/ncomms12834 PMC5052635 27670347

[55] Link K.H. , Breaker R.R. Engineering ligand-responsive gene-control elements: lessons learned from natural riboswitches. Gene Ther. 2009; 16:1189–1201. 19587710 10.1038/gt.2009.81 PMC5325117

[56] Vogel M. , Weigand J.E., Kluge B., Grez M., Suess B. A small, portable RNA device for the control of exon skipping in mammalian cells. Nucleic Acids Res. 2018; 46:E48. 29420816 10.1093/nar/gky062 PMC5934650

[57] Finke M. , Brecht D., Stifel J., Gense K., Gamerdinger M., Hartig J.S. Efficient splicing-based RNA regulators for tetracycline-inducible gene expression in human cell culture and C. elegans. Nucleic Acids Res. 2021; 49:E71. 33893804 10.1093/nar/gkab233 PMC8266659

