# Supplementary material for: Exploiting natural riboswitches for aptamer engineering and validation
Source: Nucleic Acids Res. 2023 Jan 9;51(2):966–81. doi: 10.1093/nar/gkac1218 (PMC9881172; doi:10.1093/nar/gkac1218)
Supplement: gkac1218_Supplemental_Files [file gkac1218_supplemental_files.zip › engineered_riboswitches_SuppData_S2.zip!/bbmap/pytools/template/filter_body_template.html]

Filtering is performed using BBTools rqcfilter.sh script.

## Fastq Files

|  | Number of Reads | File Size | File Location |
| --- | --- | --- | --- |
| Input FASTQ | [\_RAW-READ-CNT\_] | [\_RAW-FILE-SIZE\_] MB | [\_RAW-FILE-LOCATION\_] |
| Filtered FASTQ | [\_FILTERED-READ-CNT\_] | [\_FILTERED-FILE-SIZE\_] MB | [\_FILTERED-FILE-LOCATION\_] |
| Filtered % | [\_REMOVED-READ-PCT\_]% | - | - |

The file size for the filtered fastq is significantly smaller than the input fastq because the reads were re-ordered to optimize gzip file compression.

  

## Filtering Metrics

| Metric | Read Count | Base Count |
| --- | --- | --- |
| Input | [\_RAW-READ-CNT\_] | [\_RAW-BASE-CNT\_] |
| Remaining | [\_FILTERED-READ-CNT\_] | [\_FILTERED-BASE-CNT\_] |
| Percentage Removed | [\_REMOVED-READ-PCT\_]% | [\_REMOVED-BASE-PCT\_]% |

  

| Metric | Percent | Read Count |
| --- | --- | --- |
| Low Quality Removed | [\_LOW-QUAL-REMOVED-PCT\_] | [\_LOW-QUAL-REMOVED-READ-CNT\_] |
| Artifact Removed | [\_ARTI-REMOVED-PCT\_] | [\_ARTI-REMOVED-READ-CNT\_] |
| Ribosomal RNA Removed | [\_RRNA-REMOVED-READ-PCT\_] | [\_RRNA-REMOVED-READ-CNT\_] |
| Microbial Removed | [\_MICROBE-REMOVED-READ-PCT\_] | [\_MICROBE-REMOVED-READ-CNT\_] |
| Human Filtered | [\_HUMAN-REMOVED-READ-PCT\_] | [\_HUMAN-REMOVED-READ-CNT\_] |
| Dog Filtered | [\_DOG-REMOVED-READ-PCT\_] | [\_DOG-REMOVED-READ-CNT\_] |
| Cat Filtered | [\_CAT-REMOVED-READ-PCT\_] | [\_CAT-REMOVED-READ-CNT\_] |
| Mouse Filtered | [\_MOUSE-REMOVED-READ-PCT\_] | [\_MOUSE-REMOVED-READ-CNT\_] |

  
[\_FILTERED-READ-QC\_]
