# Supplementary material for: Exploiting natural riboswitches for aptamer engineering and validation
Source: Nucleic Acids Res. 2023 Jan 9;51(2):966–81. doi: 10.1093/nar/gkac1218 (PMC9881172; doi:10.1093/nar/gkac1218)
Supplement: gkac1218_Supplemental_Files [file gkac1218_supplemental_files.zip › engineered_riboswitches_SuppData_S2.zip!/bbmap/pytools/template/readqc_artifacts.html]

|  |  |  |
| --- | --- | --- |
| Artifacts (first [\_CONTAM-ART-FIRST-BP\_]db) | [\_CONTAM-ART-FIRST-BP-SEAL-PCT\_]% | The 1st 50bp (20bp for smRNA) of reads are aligned to the artifacts database |
