# Supplementary material for: Exploiting natural riboswitches for aptamer engineering and validation
Source: Nucleic Acids Res. 2023 Jan 9;51(2):966–81. doi: 10.1093/nar/gkac1218 (PMC9881172; doi:10.1093/nar/gkac1218)
Supplement: gkac1218_Supplemental_Files [file gkac1218_supplemental_files.zip › engineered_riboswitches_SuppData_S2.zip!/bbmap/pytools/template/readqc_body_template.html]

Read QC analysis is generated by subsampling fastq to [\_SUBSAMPLE-RATE\_]% of the reads.   
Average Base Quality, Average Read Quality, Read GC and Cycle Nucleotide Composition are generated by the BBTools reformat.sh command.

## Average Base Quality

- The table contains the percentage of bases with a given quality score.

|  |  |
| --- | --- |
| Overall Base Quality Score: | [\_BASE-QUALITY-SCORE\_] ± [\_BASE-QUALITY-SCORE-STD\_] |
| Q30 Base Quality Score: | [\_Q30-BASE-QUALITY-SCORE\_] ± [\_Q30-BASE-QUALITY-SCORE-STD\_] |

| Quality Value Threshold | ≥Q30 | ≥Q25 | ≥Q20 | ≥Q15 | ≥Q10 | ≥Q5 |
| --- | --- | --- | --- | --- | --- | --- |
| Count of Bases | [\_COUNT-OF-BAESE-Q30\_] | [\_COUNT-OF-BAESE-Q25\_] | [\_COUNT-OF-BAESE-Q20\_] | [\_COUNT-OF-BAESE-Q15\_] | [\_COUNT-OF-BAESE-Q10\_] | [\_COUNT-OF-BAESE-Q5\_] |
| Percentage % | [\_PCT-OF-BAESE-Q30\_] | [\_PCT-OF-BAESE-Q25\_] | [\_PCT-OF-BAESE-Q20\_] | [\_PCT-OF-BAESE-Q15\_] | [\_PCT-OF-BAESE-Q10\_] | [\_PCT-OF-BAESE-Q5\_] |

## Average Read Quality

- The data file contains the percentage of reads with a given average read quality.
- The plot is a histogram of average read quality.

| Quality Value Threshold | ≥Q30 | ≥Q25 | ≥Q20 | ≥Q15 | ≥Q10 | ≥Q5 |
| --- | --- | --- | --- | --- | --- | --- |
| Percentage of Reads % | [\_PCT-OF-READS-Q30\_] | [\_PCT-OF-READS-Q25\_] | [\_PCT-OF-READS-Q20\_] | [\_PCT-OF-READS-Q15\_] | [\_PCT-OF-READS-Q10\_] | [\_PCT-OF-READS-Q5\_] |

  

Average Read Quality Histogram ([\_AVG-READ-QUAL-HISTO-DATA\_], [\_AVG-READ-QUAL-HOSTO-D3\_] )

## Average Base Position Quality

- The data file contains the percentage of reads with a given average read quality.
- The plot shows average quality at each base position/cycle.

Q20 for Read 1: [\_READ\_Q20\_READ1\_]   
Q20 for Read 2: [\_READ\_Q20\_READ1\_]

Average Base Position Quality Plot ( [\_AVG-BASE-POS-QUAL-HISTO-DATA\_], [\_AVG-BASE-POS-QUAL-HISTO-D3\_] )

## Insert Size

- BBTools bbmerge.sh is used to perform this analysis. bbmerge merges paired reads into single reads by overlap detection. With sufficient coverage, bbmerge can also merge nonoverlapping reads using gapped kmers.
- The "percentage reads joined" is calculated by (number of joined reads/total number of reads \* 100).
- Raw reads are used for the insert size calculation (no trimming or filtering).

Insert Size Statistics

| Percentage Reads Joined | Average | Stddev | Mode |
| --- | --- | --- | --- |
| [\_PCT-READS-JOINED\_] | [\_PCT-READS-JOINED-AVG\_] | [\_PCT-READS-JOINED-STDDEV\_] | [\_PCT-READS-JOINED-MODE\_] |

  

Insert size histogram ( [\_INSERT-SIZE-HISTO-DATA\_], [\_INSERT-SIZE-HISTO-D3\_] )

## Read GC

- Histogram of GC percents for subsampled reads.

Average Read GC: [\_READ-GC-AVG\_] ± [\_READ-GC-STDDEV\_]%
  
  

Read GC Histogram ([\_READ-QC-HISTO-DATA\_], [\_READ-QC-HISTO-D3\_] )

## Cycle Nucleotide Composition

- The plot(s) show nucleotide counts at each cycle position.
- 1 plot is created for each read.

  

|  |  |
| --- | --- |
| Read 1 Nucleotide Composition Frequency Plot ([\_NUC-COMP-FREQ-R1-DATA\_], [\_NUC-COMP-FREQ-R1-D3\_]) | Read 2 Nucleotide Composition Frequency Plot ([\_NUC-COMP-FREQ-R2-DATA\_], [\_NUC-COMP-FREQ-R2-D3\_]) |
|  |  |

## Percentage of Common Contaminants

- The percentage of common contaminants in the randomly sampled reads are calculated using BBTools seal.sh and reported in the table.
  Phix: PhiX control sequence.

| Contaminant | Value | Contamination DB Description |
| --- | --- | --- |
| Artifacts | [\_CONTAM-ART-SEA-PCT\_]% | Illumina sequencing artifacts and adapters not including DNA or RNA spike-ins |
[\_CONTAN-ART-SEAL-FIRST-BP\_]| DNA spike-ins | [\_DNA-SPIKEIN-SEAL\_PCT\_]% | DNA spike-in sequences |
| RNA spike-ins | [\_RNA-SPIKEIN-SEAL\_PCT\_]% | RNA spike-in sequences |
| Fosmid | [\_CONTAM-FOSMID-SEAL-PCT\_]% | fosmid sequences |
| Mitochondria | [\_CONTAM-MITO-SEAL-PCT\_]% | mitochondria sequences |
| Chloroplast | [\_CONTAM-CHLO-SEAL-PCT\_]% | chloroplast sequences |
| PhiX | [\_CONTAM-PHIX-SEAL-PCT\_]% | control sequences |
| rRNA | [\_CONTAM-RRNA-SEAL-PCT\_]% | ribosomal RNA sequences |
| Non-synthetic Contaminants | [\_CONTAM-NON-SYN-SEAL-PCT\_]% | common microbial contamination found in the sequencing process |
| Synthetic Contaminants | [\_CONTAM-SYN-SEAL-PCT\_]% | synthetic sequencess used in library construction appearing as systematic contaminants |

[\_SKETCH-TABLE\_]
