# Supplementary material for: Exploiting natural riboswitches for aptamer engineering and validation
Source: Nucleic Acids Res. 2023 Jan 9;51(2):966–81. doi: 10.1093/nar/gkac1218 (PMC9881172; doi:10.1093/nar/gkac1218)
Supplement: gkac1218_Supplemental_Files [file gkac1218_supplemental_files.zip › engineered_riboswitches_SuppData_S2.zip!/bbmap/pytools/template/template.html]

 [\_PAGE-TITLE\_] 


[\_REPORT-TITLE\_]

Report for: [\_INPUT-FILE-NAME\_]

[\_REPORT-DATE\_]

[\_REPORT-BODY\_]
