# Supplementary material for: Exploiting natural riboswitches for aptamer engineering and validation
Source: Nucleic Acids Res. 2023 Jan 9;51(2):966–81. doi: 10.1093/nar/gkac1218 (PMC9881172; doi:10.1093/nar/gkac1218)
Supplement: gkac1218_Supplemental_Files [file gkac1218_supplemental_files.zip › engineered_riboswitches_SuppData_S2.zip!/bbmap/resources/tax_server.html]

JGI BBTools Taxonomy Server


- Skip To Main Content
- JGI Home

- Skip To Main Content
- Home
- Find Taxonomy Info
- Other BBTools
- Contact Us
- Statistics

# Welcome to the BBTools Taxonomy Server!

This service provides taxonomy information from NCBI taxID numbers, gi numbers, organism names, and accessions.   
Vertical bars (|) may cause problems on the command line and can be replaced by tilde (~).

- /name/homo\_sapiens will give taxonomy information for an organism name. (Names are case-insensitive and underscores are equivalent to spaces.)
- /id/9606 will give taxonomy information for an NCBI taxID.
- /gi/1234 will give taxonomy information from an NCBI gi number.
- /accession/NZ\_AAAA01000057.1 will give taxonomy information from an accession.
- /header/ will accept an NCBI sequence header such as gi|7|emb|X51700.1| Bos taurus
- /silvaheader/ will accept a Silva sequence header such as KC415233.1.1497 Bacteria;Spirochaetae;Spirochaetes
- /img/ will accept an IMG id such as 2724679250

## Benefits of using the BBTools Taxonomy Server

- Identifies all intermediate stages of taxonomy tree such as order, phylum, tribe, etc.
- Provides the information in json format
- Fast processing
- Allows queries in bulk with lists instead of single query

Examples of Basic Usage

All addresses below are assumed to be prefixed by https://taxonomy.jgi-psf.org, e.g. /name/homo\_sapiens implies a full URL of:

- https://taxonomy.jgi-psf.org/name/homo\_sapiens

For a simplified taxonomic tree, add "simple".
This will ignore unranked or uncommon levels like tribe and parvorder, and only display the following levels: SUBSPECIES, SPECIES, GENUS, FAMILY, ORDER, CLASS, PHYLUM, KINGDOM, SUPERKINGDOM, DOMAIN
For example:

- /simple/id/1234

To print taxonomy from the command line in Linux, use curl:

- curl https://taxonomy.jgi-psf.org/id/9606

Queries longer than around 8kB can be sent via POST: curl https://taxonomy.jgi-psf.org/POST...where the data sent is, for example:

- name/e.coli,h.sapiens,c.lupus

Examples of Advanced Usage

Comma-delimited lists are accepted for bulk queries, such as tax/gi/1234,7000,42
For plaintext (non-Json) results, add the term /pt/ or /sc/.
pt will give just the taxID, while sc will give the whole lineage, semicolon-delimited. For example:

- /pt/name/homo\_sapiens
- /sc/gi/1234

Additional supported display options are children, numchildren, range, simple, path, size, and ancestor.
The order is not important but they need to come before the query term. For example:

- /children/numchildren/range/gi/1234

To find the common ancestor of multiple organisms, add /ancestor/. For example:

- /id/ancestor/1234,5678,42
- /name/ancestor/homo\_sapiens,canis\_lupus,bos\_taurus

```
STATISTICSSTRING
```


- Contact Us
- Disclaimer


- Accessibility/Section 508

- © 1997-2019 The Regents of the University of California.
