# Supplementary material for: Exploiting natural riboswitches for aptamer engineering and validation
Source: Nucleic Acids Res. 2023 Jan 9;51(2):966–81. doi: 10.1093/nar/gkac1218 (PMC9881172; doi:10.1093/nar/gkac1218)
Supplement: gkac1218_Supplemental_Files [file gkac1218_supplemental_files.zip › Mohsen Breaker NAR SD Final.docx]

Supplementary Data S1

**Exploiting Natural Riboswitches for Aptamer Engineering and Validation**

Michael G. Mohsen,^1,2^ Matthew K. Midy,^3^ Aparaajita Balaji,^1^ Ronald R. Breaker^1,2,3^*

^1^ Department of Molecular, Cellular and Developmental Biology, Yale University, New Haven, CT 06520

^2^ Howard Hughes Medical Institute, Yale University, New Haven, CT 06520

^3^ Department of Molecular Biophysics and Biochemistry, Yale University, New Haven, CT 06520

* To whom correspondence should be addressed. Tel: +1 (203) 432-9389; Fax: +1 (203) 432-6161; Email: ronald.breaker@yale.edu

Table of Contents

[ADDITIONAL MATERIALS	3](#_Toc120453260)

[List of Custom Oligodeoxynucleotides	3](#_Toc120453261)

[List of Inserts for Reporter Constructs	4](#_Toc120453262)

[Instrumentation	5](#_Toc120453263)

[ADDITIONAL METHODS	6](#_Toc120453264)

[S2/S3-G0 Pool Generation	6](#_Toc120453265)

[Multiplexed Capture-SELEX with Scaffolds S2 and S3	6](#_Toc120453266)

[Quinine Reselection	7](#_Toc120453267)

[Generation of Double-Stranded DNA Templates	7](#_Toc120453268)

[Internal ^32^P Labeling of RNA	7](#_Toc120453269)

[5′ ^32^P Labeling of RNA	8](#_Toc120453270)

[SUPPLEMENTARY NOTES	8](#_Toc120453271)

[Supplementary Note 1	8](#_Toc120453272)

[Supplementary Note 2	8](#_Toc120453273)

[SUPPLEMENTAL FIGURES	10](#_Toc120453274)

[Figure S1. Elution profiling reveals ligand binding partners	10](#_Toc120453275)

[Figure S2. In-line probing of quinine aptamer Tonic	11](#_Toc120453276)

[Figure S3. In-line probing of guanine aptamer 13-2	12](#_Toc120453277)

[Figure S4. In-line probing of quinine aptamer 13-3	13](#_Toc120453278)

[Figure S5. In-line probing of quinine aptamer 13-4	14](#_Toc120453279)

[Figure S6. In-line probing of quinine aptamer 13-5	15](#_Toc120453280)

[Figure S7. Elution profile of selfish RNA Rogue	16](#_Toc120453281)

[Figure S8. In-line probing of caffeine aptamer Café	17](#_Toc120453282)

[Figure S9. In-line probing of Tonic grafted onto a guanine riboswitch fragment	18](#_Toc120453283)

[Figure S10. In-line probing of 13-3 grafted onto a guanine riboswitch fragment	19](#_Toc120453284)

[Figure S11. In-line probing of Café grafted onto a guanine riboswitch fragment	20](#_Toc120453285)

[Figure S12. Miller Assay of Tonic Quinine Riboswitch and Variants	21](#_Toc120453286)

[Figure S13. In-line probing of selfish RNA Rogue	22](#_Toc120453287)

[References	23](#_Toc120453288)

# ADDITIONAL MATERIALS

## List of Custom Oligodeoxynucleotides

| Name | nt | Sequence (5′→3′) |
| --- | --- | --- |
| Forward Primer | 35 | **TAATACGACTCACTATAGGAGGCTCTCGGGACGAC** |
| Reverse Primer | 22 | **TTACGATTGCAGCATCGGGACG** |
| Capture Oligonucleotide | 18 | **GTCGTCCCGAGAGCCATA-3′biotin** |
| S1-G0 Template | 99 | **TTACGATTGCAGCATCGGGACGACNNNNNNGGACATTTACGGTGCCCNNNNNNNNNNTGCGTGCCATATCCACGCGNNNNNNNGTCGTCCCGAGAGCCT** |
| S2-G0 Template | 99 | **TTACGATTGCAGCATCGGGACGACNNNNNNGGACATTTACGGTGCCCNNNNNNNNNNNGCGTGCCATATCCACGCNNNNNNNNGTCGTCCCGAGAGCCT** |
| S3-G0 Template | 99 | **TTACGATTGCAGCATCGGGACGACNNNNNNNNGGACATTTACGGTGCCCNNNNNNNNNNNGCGTGCCATATCCACGCNNNNNNGTCGTCCCGAGAGCCT** |
| 13-1 (Tonic) Template | 99 | **TTACGATTGCAGCATCGGGACGACGCCCGAGGACATTTACGGTGCCCGTCTTGAAGTTGCGTGCCATATCCACGCGGTATAGAGTCGTCCCGAGAGCCT** |
| 13-2 Template | 98 | **TTACGATTGCAGCATCGGGACGATGGGAAGGACATTTACGGTGCTCTTCATTATGTTGCGTGCCATATCCACGCGACTAGACGTCGTCCCGAGAGCCT** |
| 13-3 Template | 98 | **TTACGATTGCAGCATCGGGACGAGGTGAAGGACATTTACGGTGCCCTTTTCAGGCTTGCGTGCCATATCCACGCGGTATGACGTCGTCCCGAGAGCCT** |
| 13-4 Template | 99 | **TTACGATTGCAGCATCGGGACGACGCCCGAGGACATTTACGGTGCCCAAGTTGAAGTTGCGTGCCATATCCACGCGGTATACAGTCGTCCCGAGAGCCT** |
| 13-5 Template | 97 | **TTACGATTGCAGCATCGGGACGAGTAGTTGGGCATTTACGGTGCCCGGTTTATGCTTGCGTGCCATATCCACGCGAGGTCTGTCGTCCCGAGAGCCT** |
| 16-2 (Rogue) Template | 99 | **TTACGATTGCAGCATCGGGACGACTGGACGGGACATTTACGATGCCCACAAGAGCACTGCGTGCCACGTCCACGAGAGCTTCCGTCGTCCCGAGAGCCT** |
| Rogue-M1 Sense Template | 118 | **TAATACGACTCACTATAGGAGGCTCTCGGGACGACGGAAGCTATCGTGGACGTGGCACGCAGTGCTCTTGTGGGCATCGTAAATGTCCCGTCCAGTCGTCCCGATGCTGCAATCGTAA** |
| Rogue-M1 Antisense Template | 118 | **TTACGATTGCAGCATCGGGACGACTGGACGGGACATTTACGATGCCCACAAGAGCACTGCGTGCCACGTCCACGATAGCTTCCGTCGTCCCGAGAGCCTCCTATAGTGAGTCGTATTA** |
| 9-2 (Café) Template | 118 | **TAATACGACTCACTATAGGAGGCTCTCGGGACGACAGTGCACCGCGTGAATATGGCACGCAACATACCTATGGCCACCGTAAATGTCCATGTCTGACGTCCCGATGCTGCAATCGTAA** |
| Tonic with ‘Guanine Stem’ Sense Template | 109 | **TAATACGACTCACTATAGGAATATAATAGGAACACTCACTATACCGCGTGGATATGGCACGCAACTTCAAGACGGGCACCGTAAATGTCCTCGGGTGGGTGAGCAATGG** |
| Tonic with ‘Guanine Stem’ Antisense Template | 109 | **CCATTGCTCACCCACCCGAGGACATTTACGGTGCCCGTCTTGAAGTTGCGTGCCATATCCACGCGGTATAGTGAGTGTTCCTATTATATTCCTATAGTGAGTCGTATTA** |
| Tonic-M1 with ‘Guanine Stem’ Sense Template | 109 | **TAATACGACTCACTATAGGAATATAATAGGAACACTCACTATACCGCGTGGATATAACACGCAACTTCAAGACGGGCACCGTAAATGTCCTCGGGTGGGTGAGCAATGG** |
| Tonic-M1 with ‘Guanine Stem’ Antisense Template | 109 | **CCATTGCTCACCCACCCGAGGACATTTACGGTGCCCGTCTTGAAGTTGCGTGTTATATCCACGCGGTATAGTGAGTGTTCCTATTATATTCCTATAGTGAGTCGTATTA** |
| Tonic-M2 with ‘Guanine Stem’ Sense Template | 109 | **TAATACGACTCACTATAGGAATATAATAGGAACACTCACTATACCGCGTGGATATGGCACGCAACTTCAAGACGGGCACCGTAAATGTCCTCAGGTGGGTGAGCAATGG** |
| Tonic-M2 with ‘Guanine Stem’ Antisense Template | 109 | **CCATTGCTCACCCACCTGAGGACATTTACGGTGCCCGTCTTGAAGTTGCGTGCCATATCCACGCGGTATAGTGAGTGTTCCTATTATATTCCTATAGTGAGTCGTATTA** |
| 13-3 with ‘Guanine Stem’ Sense Template | 116 | **TAATACGACTCACTATAGGAATATAATAGGAACACTCAGACGTCATACCGCGTGGATATGGCACGCAAGCCTGAAAAGGGCACCGTAAATGTCCTTCACCTCTGGGTGAGCAATGG** |
| 13-3 with ‘Guanine Stem’ Antisense Template | 116 | **CCATTGCTCACCCAGAGGTGAAGGACATTTACGGTGCCCTTTTCAGGCTTGCGTGCCATATCCACGCGGTATGACGTCTGAGTGTTCCTATTATATTCCTATAGTGAGTCGTATTA** |
| Café with ‘Guanine Stem’ Sense Template | 111 | **TAATACGACTCACTATAGGAATATAATAGGAACACTCAAGTGCACCGCGTGAATATGGCACGCAACATACCTATGGCCACCGTAAATGTCCATGTCTTGGGTGAGCAATGG** |
| Café with ‘Guanine Stem’ Antisense Template | 111 | **CCATTGCTCACCCAAGACATGGACATTTACGGTGGCCATAGGTATGTTGCGTGCCATATTCACGCGGTGCACTTGAGTGTTCCTATTATATTCCTATAGTGAGTCGTATTA** |
| Tonic (mutagenized at 6% degeneracy) | 99 | **TTACGATTGCAGCATCGGGACGACGCCCGAGGACATTTACGGTGCCCGTCTTGAAGTTGCGTGCCATATCCACGCGGTATAGAGTCGTCCCGAGAGCCT** |
| 13-3 (mutagenized at 6% degeneracy) | 98 | **TTACGATTGCAGCATCGGGACGAGGTGAAGGACATTTACGGTGCCCTTTTCAGGCTTGCGTGCCATATCCACGCGGTATGACGTCGTCCCGAGAGCCT** |
| 13-5 (mutagenized at 6% degeneracy) | 97 | **TTACGATTGCAGCATCGGGACGAGTAGTTGGGCATTTACGGTGCCCGGTTTATGCTTGCGTGCCATATCCACGCGAGGTCTGTCGTCCCGAGAGCCT** |

## List of Inserts for Reporter Constructs

pDG1661-Tonic-*lysC*

**GAATTCCAAAAATAATGTTGATCCTTTTAAATAAGTCTGATAAAATGTGAACTAAAATATAATAGGAACACTCACTATACCGCGTGGATATGGCACGCAACTTCAAGACGGGCACCGTAAATGTCCTCGGGTGGGTGAGCAATGGAACCGCACGTGTACGGTTTTTTGTGATATCAGCATTGCTTGCTCTTTATTTGAGCGGGCAATGCTTTTTTTATTCTCATAACGGAGGTAGACAGGATGGAAGCACTGAGGATCC**

pDG1661-Tonic-M1*-lysC*

**GAATTCCAAAAATAATGTTGATCCTTTTAAATAAGTCTGATAAAATGTGAACTAAAATATAATAGGAACACTCACTATACCGCGTGGATATAACACGCAACTTCAAGACGGGCACCGTAAATGTCCTCGGGTGGGTGAGCAATGGAACCGCACGTGTACGGTTTTTTGTGATATCAGCATTGCTTGCTCTTTATTTGAGCGGGCAATGCTTTTTTTATTCTCATAACGGAGGTAGACAGGATGGAAGCACTGAGGATCC**

pDG1661-Tonic-M2*-lysC*

**GAATTCCAAAAATAATGTTGATCCTTTTAAATAAGTCTGATAAAATGTGAACTAAAATATAATAGGAACACTCACTATACCGCGTGGATATGGCACGCAACTTCAAGACGGGCACCGTAAATGTCCTCAGGTGGGTGAGCAATGGAACCGCACGTGTACGGTTTTTTGTGATATCAGCATTGCTTGCTCTTTATTTGAGCGGGCAATGCTTTTTTTATTCTCATAACGGAGGTAGACAGGATGGAAGCACTGAGGATCC**

pDG1661-Café*-lysC*

**GAATTCCAAAAATAATGTTGATCCTTTTAAATAAGTCTGATAAAATGTGAACTAAAATATAATAGGAACACTCAAGTGCACCGCGTGAATATGGCACGCAACATACCTATGGCCACCGTAAATGTCCATGTCTTGGGTGAGCAATGGAACCGCACGTGTACGGTTTTTTGTGATATCAGCATTGCTTGCTCTTTATTTGAGCGGGCAATGCTTTTTTTATTCTCATAACGGAGGTAGACAGGATGGAAGCACTGAGGATCC**

pDG1661-Café-M1*-lysC*

**GAATTCCAAAAATAATGTTGATCCTTTTAAATAAGTCTGATAAAATGTGAACTAAAATATAATAGGAACACTCAAGTGCACCGCGTGAATATAACACGCAACATACCTATGGCCACCGTAAATGTCCATGTCTTGGGTGAGCAATGGAACCGCACGTGTACGGTTTTTTGTGATATCAGCATTGCTTGCTCTTTATTTGAGCGGGCAATGCTTTTTTTATTCTCATAACGGAGGTAGACAGGATGGAAGCACTGAGGATCC**

pDG1661-Café-M2*-lysC*

**GAATTCCAAAAATAATGTTGATCCTTTTAAATAAGTCTGATAAAATGTGAACTAAAATATAATAGGAACACTCAAGTGCACCGCGTGAATATGGCACGCAACATACCTATGGCCACCGTAAATGTCCATATCTTGGGTGAGCAATGGAACCGCACGTGTACGGTTTTTTGTGATATCAGCATTGCTTGCTCTTTATTTGAGCGGGCAATGCTTTTTTTATTCTCATAACGGAGGTAGACAGGATGGAAGCACTGAGGATCC**

**Key: EcoRI / *lysC* promoter / guanine riboswitch (transcription start site and start codon underlined) / test aptamer / mutation(s) / BamHI**

## Instrumentation

Eppendorf Nexus Thermal Cycler (Mastercycler Nexus Gx2, Mastercycler Nexus Gx2e, Mastercycler Nexus Gx2e) was used for thermal cycling. Bio-Rad CFX Opus RT-PCR System (CFX Opus 96) was used for quantitative PCR (qPCR). BioTek Synergy Neo2 Multimode Reader was used for quantitative β-galactosidase assay. Varian Cary 60 was used to measure optical density (OD) at 600 nm. Typhoon FLA9500 was used for phosphor imaging. Gel Doc Go Imaging System (Bio-Rad) was used to image agarose gels stained with ethidium bromide. TriCarb 4910 scintillation counter was used in some cases to quantify radioactivity levels of eluates collected during an elution profile. NanoDrop 8000 (Thermo Fisher) was used to measure the concentration of solutions containing nucleic acids.

# ADDITIONAL METHODS

## S2/S3-G0 Pool Generation

The S2/S3-G0 pool was prepared similarly to the S1-G0 pool, with a few minor differences. The overlap extension reaction contained 75 pmol each of S2-G0 Template and S3-G0 Template, and 150 pmol Forward Primer. A single in vitro transcription reaction containing one-fourth of the overlap extension reaction (25 pmol) was incubated at 37 °C for 2 h. The RNA pool was purified without further changes to the protocol described above.

**Preparation of Selection Buffer**

4.77 g HEPES, 50 mL 2M KCl, 1 mL 1 M MgCl_2_, and 35 mL water were mixed in a flask containing a magnetic stir bar. The pH of the solution was adjusted to 7.4 by adding NaOH pellets while stirring (pellets were weighed before adding). Once pH 7.4 was attained, any remaining pellets were removed, dried, and weighed. After calculating the amount of NaOH required to adjust the pH (approximately 70 mM), a volume (approximately 0.6 mL) of 5 M NaCl was added to the solution to bring the total [Na^+^] to 100 mM. Water was added to adjust the final volume of the solution to 100 mL, and the resulting solution was filter-sterilized. The final solution is a 10x concentrated selection buffer containing 200 mM HEPES, 1 M K^+^, 100 mM Na^+^, 10 mM Mg^2+^, 1.05 M Cl^-^, pH 7.4 at ~20°C (1x: 20 mM HEPES, 100 mM K^+^, 10 mM Na^+^, 1 mM Mg^2+^, 105 mM Cl^-^).

## Multiplexed Capture-SELEX with Scaffolds S2 and S3

The protocol was performed as described for rounds 14-16 with Scaffold S1, except that the washing step was modified to increase stringency. After applying the annealed RNA-capture oligonucleotide solution to the column three times, the column was washed six times with 100 µL selection buffer. For these six washes, the buffer was loaded onto the column and the air pressure control device was used to immediately drain the liquid into a collection tube. The purpose of these washes is to remove nonspecifically bound RNA molecules from the column. Next, 100 µL selection buffer was loaded onto the column, and the air pressure control device was used to push the solution into the matrix, but not through the column, such that the matrix is incubated in the solution. The matrix is incubated for 2.5 min before the solution is pushed through with the air pressure control device. The purpose of this step is a negative selection against RNA sequences that displace from the capture oligonucleotide in a time-dependent manner. Subsequent steps were performed without modification to the protocol described above for rounds 14-16 with Scaffold S1. For selection with Scaffold S2, 100 pmol of RNA pool was used in rounds 1-2, and 40 pmol was used in rounds 3-9, with a 5x molar excess of capture oligonucleotide in all cases.

## Quinine Reselection

For the design of the mutagenized oligodeoxynucleotide templates, the region in between the two primer-binding regions was mutagenized by custom-order synthesis. Four custom nucleotide mixes were used: (94% A, 2% T, 2% G, 2% C), (2% A, 94% T, 2% G, 2% C), (2% A, 2% T, 94% G, 2% C), and (2% A, 2% T, 2% G, 94% C). Wherever an instance of a given nucleotide was present in the original sequence, this nucleotide was replaced with the mix containing 94% of that nucleotide in the synthesis of the mutagenized pool. The three resulting oligonucleotides were mixed in a 1:1:1 ratio (33.3 pmol each) and the dsDNA pool was prepared by overlap extension with the Forward Primer. The remaining pool generation steps were similar to those described above for other pools. Capture-SELEX was performed similarly to rounds 14-16 with Scaffold S1, with a few exceptions. Instead of a collection of compounds, a solution containing only quinine (1 µM) in selection buffer was used. Five rounds of selection were performed.

## Generation of Double-Stranded DNA Templates

Three methods were employed to generate dsDNA templates containing a T7 promoter, which were subsequently used for in vitro transcription. One method involved overlap extension of the reverse complement of the desired sequence (which excluded the T7 promoter sequence) and the Forward Primer. This was performed similarly to the pool generation step described above. dsDNA templates for Tonic, 13-2 through -5, and Rogue were produced using this method. An alternative method was used to generate dsDNA templates of Café, which involved performing 20 cycles of PCR with the full-length single-stranded DNA template, Forward Primer, and Reverse Primer. A third method was used to generate all other dsDNA templates in which the corresponding full-length sense and antisense oligonucleotides were mixed and heated at 90 °C for 3-5 min then allowed to cool to room temperature. All dsDNA templates were used without further purification for in vitro transcription with T7 RNA polymerase to produce RNA transcripts of the templated sequence.

## Internal ^32^P Labeling of RNA

A 20 µL reaction mix was prepared containing 2 pmol of a dsDNA template, transcription buffer, 2 mM NTPs, T7 RNA polymerase, and 10 µCi of α-^32^P-ATP. The reaction was incubated at 37 °C for 2 h. Internally labeled RNA was crudely purified using a gel filtration cartridge. Before loading the reaction mixture, the gel filtration cartridge was centrifuged at 800 x g for 1 min to drain the storage buffer. The cartridge was placed in a new collection tube and the entire reaction mixture was loaded onto a gel filtration cartridge. The cartridge was centrifuged at 800 x g for 1 min to recover the RNA.

## 5′ ^32^P Labeling of RNA

A 100 µL reaction mix was prepared containing 25 pmol dsDNA template, 2 mM NTPs, transcription buffer, and T7 RNA polymerase. The reaction was incubated at 37 °C for 2 h, or until the formation of a white precipitate. The reaction was quenched by the addition of 100 µL loading buffer and purified by denaturing 10% PAGE. The band containing RNA of the desired length was visualized by UV shadowing and excised from the gel. The RNA was recovered by crushing and soaking the gel piece in elution buffer (200 mM NaCl, 10 mM Tris-HCl [pH 7.5 at ~20 °C], 1 mM EDTA [pH 8 at ~20 °C]), followed by precipitation in ethanol. A 20 µL reaction mix was prepared containing 75 pmol of RNA, rAPid reaction buffer, and rAPid alkaline phosphatase. The reaction was incubated at 37 °C for 1 h. One-fifth of this reaction (15 pmol) was transferred to a new tube. T4 polynucleotide kinase reaction buffer, T4 polynucleotide kinase, 10 µCi γ-^32^P-ATP and water to 20 µL were added and the reaction was incubated at 37 °C for 1 h. Then, 20 µL of loading buffer was added to this solution. 5′ ^32^P-labeled RNA was purified by denaturing 10% PAGE. The band containing RNA of the desired length was visualized by phosphor imaging. RNA was recovered from the excised gel piece as described above.

# SUPPLEMENTARY NOTES

## Supplementary Note 1

We often conduct preliminary or ‘scout’ in-line probing assays that reveal ligand induced structural changes and approximate *K*_D_ values. We then design follow-up experiments to generate the datasets that are presented in the main and supplemental texts. Each analysis is thus repeated several times, and a single representative gel and binding curve is presented for each in-line probing analysis. A recent report (1) verifies that *K*_D_ values estimated by in-line probing are in good agreement with those estimated by other methods, including isothermal titration calorimetry (ITC) and surface plasmon resonance (SPR).

## Supplementary Note 2

Our first selection campaign generated trillions of copies of Rogue through repeated amplification. Inadvertent contamination might have occurred through aerosolization of microdroplets containing this sequence during sample manipulation or due to spreading by surface contact. The same PCR primers were used in both the original and new selection lines during amplification. Thus, it appears likely that a substantial amount of Rogue contaminated the S2/S3 selection line at an early stage, perhaps during the pool generation step.

# SUPPLEMENTAL FIGURES

## Figure S1. Elution profiling reveals ligand binding partners

**A.** Elution profile (n = 1) with internally ^32^P-labeled S1-G13 pool. Eluates resulting from incubation with solutions containing ligand candidates are shown. The elution order follows sequentially from left to right. The radioactivity contained in each eluate was quantitated using a scintillation counter and normalized to the highest signal (set to 100%). **B.** Elution profiles (n = 1) with internally (Tonic and 13-2 through -5) or 5′ (Café) ^32^P-labeled aptamer candidate sequences. For Tonic and 13-3, radioactivity levels were quantitated using a scintillation counter and normalized to the highest signal. For all other samples, eluates were analyzed by dot blot analysis. Relative levels of radioactivity contained in each eluate were assessed by densitometry.

## Figure S2. In-line probing of quinine aptamer Tonic

**A.** In-line probing analysis with each of the eight compounds (10^-4^ M) from the collection. **B.** Secondary structure model of Tonic RNA, including paired elements P1, P2, and P3 as well as a putative pseudoknot (*pk*), supported by in-line probing analysis. **C.** In-line probing analysis with various concentrations of quinine ranging from 10^-8^ M to 10^-4^ M at quarter-log intervals. Three modulating sites (called 1, 2, and 3) were quantitated by densitometry. **D.** To estimate the *K_D_* of this interaction, the fraction of bound RNA was calculated from the three modulating sites and plotted *vs.* the logarithm of concentration.

## Figure S3. In-line probing of guanine aptamer 13-2

**A.** In-line probing analysis of 13-2 RNA with various concentrations of guanine ranging from 10^-8^ M to 10^-4^ M at log intervals, and each of the seven other compounds from the collection (10^-4^ M). **B.** Secondary structure model of 13-2 RNA, including paired elements P1, P2, and P3 as well as a putative pseudoknot (*pk*), supported by in-line probing analysis.

## Figure S4. In-line probing of quinine aptamer 13-3

**A.** In-line probing analysis with various concentrations of quinine ranging from 10^-8^ to 10^-4^ M at log intervals and 10^-3.5^ M, and each of the other seven compounds (10^-4^ M) from the collection. **B.** Secondary structure model of 13-3 RNA, including paired elements P1, P2, and P3 as well as a putative pseudoknot (*pk*), supported by in-line probing analysis. **C.** In-line probing analysis with various concentrations of quinine ranging from 10^-8^ M to 10^-4^ M at quarter-log intervals. Three modulating sites were quantitated by densitometry. **D.** To estimate the *K_D_* of this interaction, the fraction of bound RNA was calculated from the three modulating sites and plotted *vs.* the logarithm of concentration.

## Figure S5. In-line probing of quinine aptamer 13-4

**A.** In-line probing analysis of 13-4 RNA with various concentrations of quinine ranging from 10^-8^ M to 10^-4^ M at log intervals and 10^-3.5^ M, and each of the seven other compounds from the collection (10^-4^ M). **B.** Secondary structure model of 13-4 RNA, including paired elements P1, P2, and P3 as well as a putative pseudoknot (*pk*), supported by in-line probing analysis.

## Figure S6. In-line probing of quinine aptamer 13-5

**A.** In-line probing analysis of 13-5 RNA with various concentrations of quinine ranging from 10^-8^ M to 10^-4^ M at quarter-log intervals. **B.** Secondary structure model of 13-5 RNA, including paired elements P1, P2, and P3 as well as a putative pseudoknot (*pk*), supported by in-line probing analysis. **C.** To estimate the *K_D_* of this interaction, the fraction of bound RNA was calculated from the three modulating sites and plotted *vs.* the logarithm of concentration.

## Figure S7. Elution profile of selfish RNA Rogue

Elution profile (n = 1) with ^32^P-labeled Rogue. Eluates resulting from incubation with solutions containing ligand candidates (100 µM each) are shown. The elution order follows sequentially from left to right. The radioactivity contained in each eluate was quantitated using a scintillation counter and normalized to the highest signal.

## Figure S8. In-line probing of caffeine aptamer Café

**A.** In-line probing analysis with ligand candidates from the six-compound collection (10^-3^ M). **B.** Secondary structure model of Café supported by in-line probing analysis. **C.** In-line probing analysis with various concentrations of caffeine ranging from 10^-8^ M to 10^-3.75^ M at quarter-log intervals. Two modulating sites were quantitated by densitometry. **D.** Plot of fraction of bound RNA *vs.* the logarithm of concentration of caffeine.

## Figure S9. In-line probing of Tonic grafted onto a guanine riboswitch fragment

**A.** In-line probing analysis of an RNA construct designed by grafting the aptameric core of Tonic onto the P1 stem of the *B. subtilis xpt-pbuX* guanine riboswitch with various concentrations of quinine ranging from 10^-6^ M to 10^-4^ M at log intervals, and guanine at 10^-4^ M. **B.** In-line probing analysis of variants Tonic-M1 (left) and Tonic-M2 (right) with various concentrations of quinine ranging from 10^-6^ M to 10^-4^ M at log intervals, and guanine at 10^-4^ M. **C.** Secondary structure model supported by in-line probing analysis.

## Figure S10. In-line probing of 13-3 grafted onto a guanine riboswitch fragment

**A.** In-line probing analysis of an RNA construct designed by grafting the aptameric core of 13-3 onto the P1 stem of the *B. subtilis xpt-pbuX* guanine riboswitch with various concentrations of quinine ranging from 10^-6^ M to 10^-4^ M at log intervals, and guanine at 10^-4^ M. **B.** Secondary structure model supported by in-line probing analysis.

## Figure S11. In-line probing of Café grafted onto a guanine riboswitch fragment

**A.** In-line probing analysis of an RNA construct designed by grafting the aptameric core of Café onto the P1 stem of the *B. subtilis xpt-pbuX* guanine riboswitch with various concentrations of caffeine ranging from 10^-6^ M to 10^-3^ M at log intervals, and guanine at 10^-4^ M. **B.** Secondary structure model supported by in-line probing analysis.

## Figure S12. Miller Assay of Tonic Quinine Riboswitch and Variants

Results of a Miller assay performed with *B. subtilis* cells carrying the indicated constructs, cultured in media supplemented with the indicated concentrations of quinine. Each point represents data from one technical replicate, and the thin line indicates the mean.

## Figure S13. In-line probing of selfish RNA Rogue

**A.** In-line probing analysis of Rogue with each ligand candidate from the six-compound collection at a concentration of 10^-3^ M. **B.** Secondary structure model of Rogue, including paired elements P1, P2, and P3, supported by in-line probing analysis.

# References

1. Arney,J.W. and Weeks,K.M. (2022) RNA-ligand interactions quantified by surface plasmon resonance with reference subtraction. *Biochemistry*, **61**, 1625–1632.
